# Photodynamic Biomimetic Nanoparticles Accelerate Tumor Vascular Normalization Initiation

**DOI:** 10.1002/EXP.20240333

**Published:** 2026-06-05

**Authors:** Yufei Liu, Changheng Xie, Yanfeng Huang, Ting Wang, Shi Du, Hui Xiong, Jing Yao

**Affiliations:** ^1^ Jiangsu Key Laboratory of Druggability of Biopharmaceuticals Department of Pharmaceutics China Pharmaceutical University Nanjing China; ^2^ International College of Pharmaceutical Innovation Soochow University Suzhou P. R. China; ^3^ Department of Biomedical Informatics College of Medicine The Ohio State University Columbus Ohio USA

**Keywords:** biomimetic nanoparticle, photodynamic therapy, tumor vascular normalization

## Abstract

The abnormal tumor vascular networks fuel the tumor growth and aggravate the tumor hypoxia. Although vascular normalization therapy (VNT) is clinically effective, the long period before vascular normalization window (VNW) initiation presents significant prognostic challenges. Here, we develop a platelet‐mimetic nanosystem, IA@PM, which is self‐assembled by apatinib (APA) and indocyanine green and further coated with platelet membrane (PM). IA@PM accelerates the pro‐angiogenic and anti‐angiogenic factors’ balance to significantly expedite the VNW initiation of APA from the fourth day to the second day post‐treatment through rationally utilizing photodynamic therapy (PDT). Benefiting from this VNW initiation acceleration, a timelier and facilitated tumor drug delivery is achieved. This process further constructs a self‐amplified therapeutic cycle between VNT and PDT, contributing to the excellent antitumor effects. Collectively, IA@PM breaks key limiting factors in current VNT efficacy by accelerating VNW initiation and also significantly promotes PDT antitumor efficacy with lower doses of ICG, bringing revolutionary new strategies in VNT.

## Graphical abstract

Platelet‐mimetic IA@PM integrates apatinib‐driven vascular normalization with ICG‐mediated photodynamic therapy, creating a self‐amplifying vascular normalization therapy (VNT)–photodynamic therapy (PDT) cycle that accelerates the onset of the vascular normalization window and potentiates antitumor treatment.

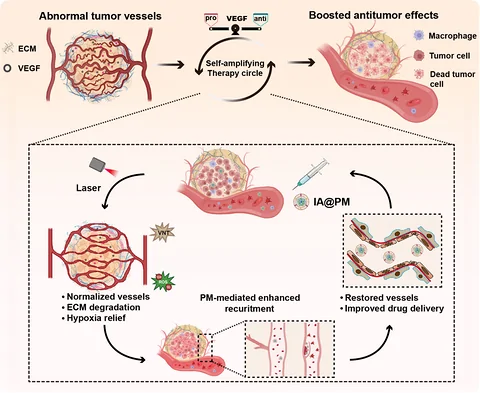

## 1 Introduction

Tumor blood vessels respond to upregulated angiogenic factors, such as pro‐angiogenic factors vascular endothelial growth factor (VEGF), forming abnormal vascular networks [1, 2]. These abnormal tumor vessels supply nutrients for tumor progression and impair oxygen delivery, causing hypoxic tumor microenvironment (TME) and finally attributing to poor therapeutic prognosis [1, 2]. Vascular normalization therapy (VNT), based on the rational utility of anti‐angiogenic drugs (e.g., VEGF inhibitors), was clinically effective in reshaping the abnormal vascular system, which inhibits tumor growth, relieves hypoxia, and improves intratumoral drug delivery [2, 3, 4, 5]. However, VEGF inhibitors, such as apatinib (APA), typically require 4–7 days of repeated administration before initiating vascular normalization window (VNW) [6, 7]. This delay in VNW initiation critically compromises treatment efficacy, especially for aggressive malignancies. For example, before VNW initiation, the melanoma volume surges over 10 times the initial volume, while intratumoral VEGF levels increase to even five times that of the original levels [7]. This dramatic increase in tumor size and VEGF concentration undermines the effectiveness of VNT and leads to a worsened prognosis. In this context, achieving earlier VNW initiation could significantly mitigate these risks by reducing tumor progression before VNT, thereby improving the overall therapeutic outcome.

The key to swiftly establishing a balance between pro/anti‐angiogenic factors for VNW initiation lies in rapidly counteracting the upregulated pro‐angiogenic factors in TME [4]. Reactive oxygen species (ROS) have been reported to act as a critical mediator in regulating tumor vessels by downregulating VEGF expression [8]. Concurrently, moderate ROS could directly and selectively damage the immature neovessels [9, 10], which has sparse pericyte coverage and exposed endothelial cells. Additionally, it could directly oxidatively degrade dense extracellular matrix (ECM), relieving intratumoral solid stress and avoiding the upregulation of ECM‐induced pro‐angiogenetic factors [11, 12, 13, 14, 15]. Inspired by these effects of ROS in rapidly balancing the pro/anti‐angiogenic factors, we propose that photodynamic therapy (PDT) could exhibit great potential in accelerating VNW initiation. PDT offers instantaneous and controlled generation of ROS, and its short half‐life avoids excessive vascular pruning [16]. However, leveraging PDT to accelerate VNW initiation still poses an advanced request in tumor targeting due to the limited diffusion distance of ROS.

**SCHEME 1:**
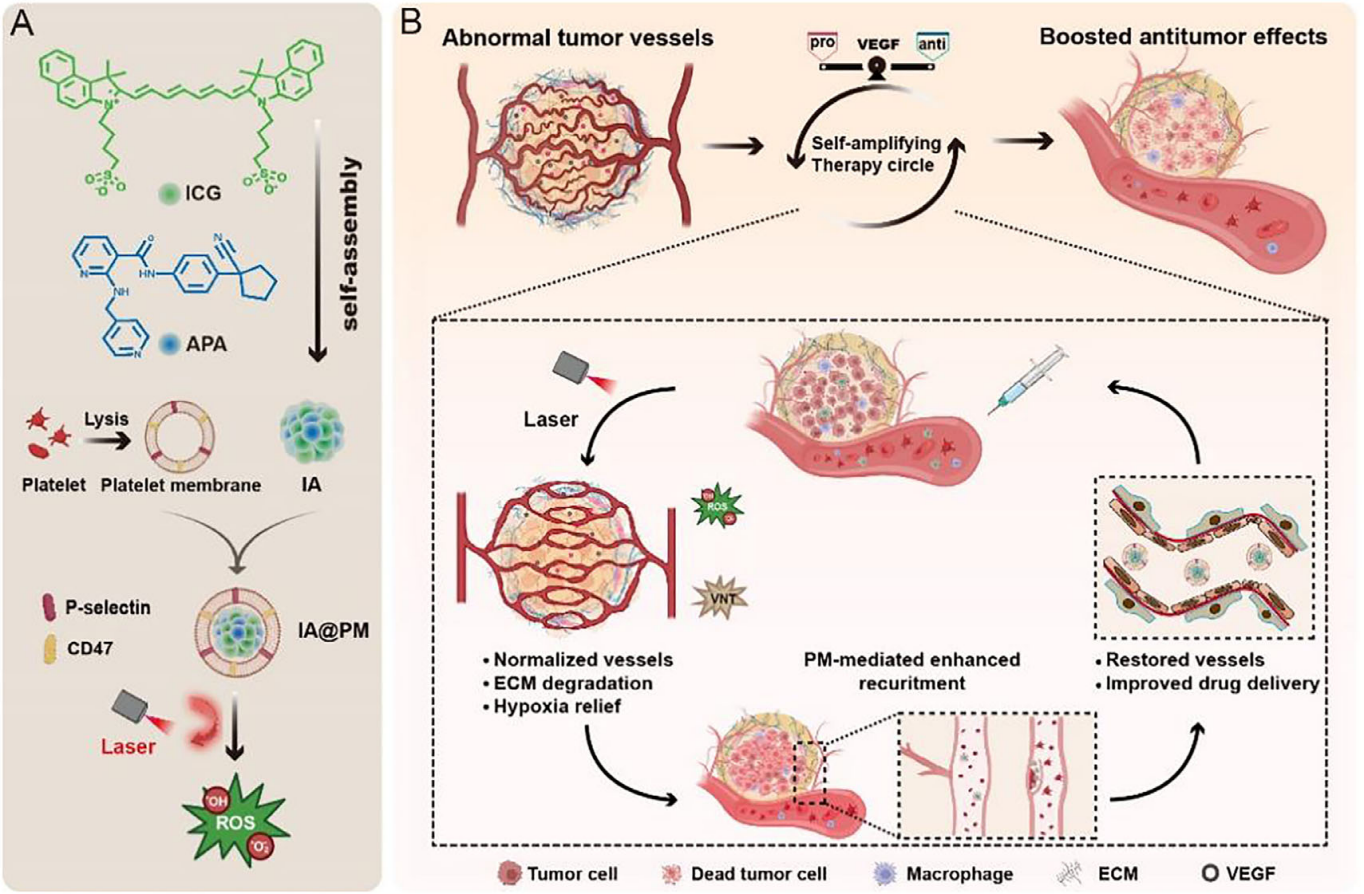
Schematic illustrations of synthetic procedure and the therapeutic mechanism of platelet‐mimetic nanosystem IA@PM. (A) The self‐assembly processes of IA@PM. (B) After intravenous injection (*i.v*.) and laser irradiation, IA@PM synergistically advanced the initiation of VNT and induced boosted antitumor effects.

To address the challenge of limited ROS diffusion while harnessing the potential of PDT in accelerating VNW initiation, we developed a biomimetic nanosystem, IA@PM, to accelerate APA‐mediated VNW initiation, which could generate moderate ROS for VNT. IA@PM was self‐assembled by the VEGFR2 inhibitor APA and the photosensitizer indocyanine green (ICG), which was further coated with platelet membrane (PM) (Scheme 1A). After combined with PDT, IA@PM employed a self‐amplifying cycle, which significantly advanced VNW (from the fourth day to the second day) initiation and enhanced its antitumor efficacy (Scheme 1B). In detail, after injection, the PM coating enabled IA@PM to evade immune surveillance and the nanoparticles circulate with blood flow to reach tumor vessels [17, 18]. Under laser irradiation, IA@PM‐mediated controlled PDT rapidly generates ROS to selectively prune abnormal tumor vessels and downregulate VEGF levels. This effect synergistically supports APA in promoting vascular normalization [19, 20, 21]. As vascular normalization progresses, the improved vessel function further facilitated both IA@PM delivery and oxygen transport deeper into the tumor. This enhanced oxygen availability extends ROS diffusion distance and mitigates PDT‐induced hypoxia, creating a positive feedback loop between VNT and PDT efficacy.

Collectively, by leveraging controlled PDT, IA@PM significantly accelerated VNW initiation and prolonged its duration. These biomimetic nanoparticles not only address the clinical challenge of delayed VNW initiation associated with anti‐VEGF treatments but also reveal a novel role for ROS in VNT. This innovative approach provides an innovative strategy to improve vascular‐targeted therapies, offering a promising direction for hypervascular solid tumor treatment.

## 2 Materials and Methods

### 2.1 Materials

APA was obtained from Jiangsu Hengrui medicine Co., Ltd. (Jiangsu, China); DPBF and ICG (purity 95%) were purchased from Aladdin Reagent (Shanghai, China); Protease inhibitors were purchased from Sigma Aldrich Trading Co., Ltd. (Shanghai, China); CD47 monoclonal antibody and P‐selectin monoclonal antibody were purchased from Sanying Biotechnology Co., Ltd. (Wuhan, China); [Ru(dpp)_3_]Cl_2_ was purchased from Maokang Biotechnology Co., Ltd. (Shanghai, China). Glucose kit, ALT kit, and AST kit were supplied by Jiancheng Bioengineering Institute (Nanjing, China). TRITC‐Dextran was from Maokang Biotechnology Co., Ltd. (Shanghai, China). Cell lines were from Nanjing KeyGEN Biotech Co., Ltd. (Nanjing, China). BALB/c (female, 6 weeks) and C57BL/6 mice (female, 6 weeks) were bought from Jiangsu Huachuang Xinnuo Pharmaceutical Technology Co., Ltd. (Jiangsu, China).

### 2.2 Preparation and Characterization of IA@PM

First, APA methanol solution (5 mg mL^−1^) and ICG aqueous solutions (0.2 mg mL^−1^) were mixed and stirred for 10 min. Then, the mixture was dialyzed and centrifuged to obtain supernatant, which was IA. The preparation of PM involved a two‐step process. First, platelet cells were obtained from whole blood and lysed using a freeze–thaw method. Then, PM was prepared through sonicating and extruding ultrasonic. The IA@PM was made by mixing PM and IA (1:1) at the conditions of sonicating. To determine drug loading (DL), IA nanoparticles were disrupted using a DMSO solution (DMSO:H_2_O = 9:1), and the resulting solution was analyzed to quantify the concentrations of ICG and APA for calculating their respective DL values. Specifically, the DL of ICG and APA were calculated as the ratio of the weight of ICG or APA in the nanoparticles to the total weight of the nanoparticles. The particle size, PDI, and zeta potential of different nanoparticles were measured with dynamic light scattering (DLS) (Brookhaven Instruments Corporation, Omni, USA). Transmission electron microscope (TEM; HITACHI, H‐7650) was employed to observe the morphology of different nanoparticles. Moreover, the drug release behavior of IA@PM was tested by dialysis method at 37°C for 24 h. To investigate the potential impact of glutathione (GSH) on the drug release characteristics of IA@PM, 1 and 10 mM GSH was added to the phosphate buffer medium (pH 7.4). Samples were collected at 0, 4, 8, 12, and 24 h, and HPLC was employed to determine the drug concentration.

### 2.3 The Assessment of Tumor Targeting Properties of IA@PM

HUVEC, B16F10, and RAW 264.7 cells were incubated with free ICG, IA, and IA@PM. After 4, 8, 12 h, the APA concentration in different cells was measured by HPLC method or flow cytometry (FCM; FACSCelesta). Furthermore, co‐culture cell models consisting of HUVEC, B16F10, and RAW 264.7 were employed to further investigate and validate the differential affinity of IA@PM for these distinct cell types. After adding IA@PM into the co‐culture cell models for 12 h, the IA@PM fluorescence intensity in different cell models was detected by FCM. The in vivo tumor targeting ability of IA@PM was validated by in vivo imaging analysis. B16F10‐bearing mice (tumor volume around 400 mm^3^) were injected with IA or IA@PM (3 mg ICG kg^−1^). At 2, 8, and 24 h after injection, DAMP fluorescent images of the mice were obtained by in vivo imaging system (IVIS Spectrum, USA). Finally, the mice were sacrificed and the major organs and tumors of every mouse were collected and imaged.

### 2.4 The Assessment of Cytotoxicity, Intracellular ROS Generation, and Cell Migration Inhibition Abilities of IA@PM

The 3‐(4,5‐dimethylthiazol‐2‐yl)‐2,5‐diphenyltetrazolium bromide (MTT) assay method was used to measure the cell cytotoxicity of IA@PM. HUVEC and B16F10 cells were incubated in 96‐well plates for 24 h with APA, ICG, IA, and IA@PM at varying concentrations for 12 h, which were irradiated (808 nm, 0.8 W cm^−^^2^, 2 min) and incubated for another 12 h. Afterward, MTT solution was added to each well and the cells were incubated for another 4 h at 37°C. The optical density (OD) at 490 nm was measured by a microplate reader (Thermo Fisher Scientific Inc., USA). ROS production in HUVEC and B16F10 cells was evaluated with 2′,7′‐dichlorofluorescein diacetate (DCFH‐DA) method. After treating the cells with ICG, IA, and IA@PM (each equivalent to 6 µg ICG mL^−1^) for 12 h, DCFH‐DA was added, followed by laser irradiation (0.8 W cm^−^^2^, 5 min). The ROS levels were detected by DCFH‐DA method. The cell migration inhibition ability of IA@PM was studied via a scratch wound healing assay. First, HUVEC cells were treated with different formulations (equivalent to 3 µg ICG mL^−1^) and irradiated with an 808 nm laser (0.8 W cm^−^^2^, 5 min). Cell migration rate was calculated according to the following equation.

(1) $$\mathrm{Cell}\;\mathrm{migration}\;\mathrm{rate}\;(\%)=\frac{\mathrm{Are}a_{0\;h}\;-\mathrm{Are}a_{24\;h}}{\mathrm{Are}a_{0\;h}}\;\;\times 100\%$$

Area_0 h_ was the initial scratch area, Area_24 h_ was the scratch area after 24 h treatment of different formulations.

### 2.5 The Evaluation of VNW Initiation

A subcutaneous melanoma tumor mouse model was employed to investigate accelerated vascular normalization. Once the tumor size reached 50 mm^3^, the melanoma‐bearing mice were randomly assigned to three distinct groups: control (saline), IA@PM, and IA@PM + Laser. The IA@PM and IA@PM + Laser groups received treatments equivalent to APA at 20 mg kg^−1^ and ICG at 2.9 mg kg^−1^. In the group of IA@PM + Laser, the mice were further irradiated with an 808 nm laser (0.6 W cm^−^^2^, 5 min). Different formulations were administered intravenously to the mice on days 0 and 2, respectively. Mice in the group requiring irradiation were irradiated at 24 h after administration. Tumor tissues were collected and analyzed for vessel morphology (using CD31 and α‐SMA), hypoxia (assessed by HIF‐1α and VEGF), and collagen content through immunofluorescence and Masson staining. Besides, the orthotopic 4T1 breast tumor mouse model was also employed to investigate the VNW initiation effect and mechanisms of IA@PM. These mice were randomly assigned to four distinct groups: control (saline), ICG + Laser (2.9 mg kg^−1^), IA@PM, and IA@PM + Laser group (APA at 20 mg kg^−1^ and ICG at 2.9 mg kg^−1^). In contrast to the experimental protocol for the melanoma tumor mouse model, orthotopic 4T1 breast tumor‐bearing mice were administered a single dose on day 0. On day 2 post‐administration, in vivo blood vessel imaging was conducted using a two‐photon laser scanning microscope (Bergamo II, Thorlabs, USA). To label the blood vessels, these 4T1 tumor‐bearing mice were injected with TRITC‐Dextran. Additionally, tumor tissues were collected to assess VEGF levels.

Animals used in this study were purchased from Jiangsu Huachuang Xinnuo Pharmaceutical Technology Co., Ltd. and maintained in China Pharmaceutical University Laboratory Animal Center. All the animals were treated according to the Guide for Care and Use of Laboratory Animals, approved by the Animal Experimentation Ethics Committee of China Pharmaceutical University. The approval number is “2025‐01‐053.”

### 2.6 The Evaluation of Antitumor Efficacy and VNT Promotion Effects of IA@PM

Once the tumor size reached 50 mm^3^, the melanoma‐bearing mice were randomly assigned to five distinct groups: control (saline), ICG + Laser (2.9 mg kg^−1^), IA + Laser, IA@PM, and IA@PM + Laser. In the group of IA + Laser, IA@PM, and IA@PM + Laser, the dose of APA was 20 mg kg^−1^ and the dose of ICG was 2.9 mg kg^−1^. Different formulations were administered intravenously to the mice on days 0, 2, 4, 6, and 8, respectively. Mice in the group requiring irradiation were further irradiated with an 808 nm laser (0.6 W cm^−^^2^, 5 min) at 24 h after administration. The body weights and the tumor volumes of all the mice were measured and documented daily. On day 14, all mice from each group were sacrificed, the tumors were excised and weighed. Following that, tumor tissue was collected for pathological analysis. Under the above experimental protocol, tumor tissues from the mice on day 8 were also collected for tumor vascular morphology assessment and hypoxia detection. The tumor vascular endothelial cells (VECs) and pericytes were stained with CD31 and α‐SMA, respectively. The areas of tumor hypoxia were stained with hypoxyprobe‐1 kits hypoxia.

### 2.7 Statistical Analysis

All statistical analyses were performed using GraphPad Prism version 8 software. One‐way analysis of variance (ANOVA) was used for statistical analysis. *p* < 0.05 was considered statistically significant between the datasets.

## 3 Results and Discussion

### 3.1 Preparation and Characterization of IA and IA@PM

**FIGURE 1:**
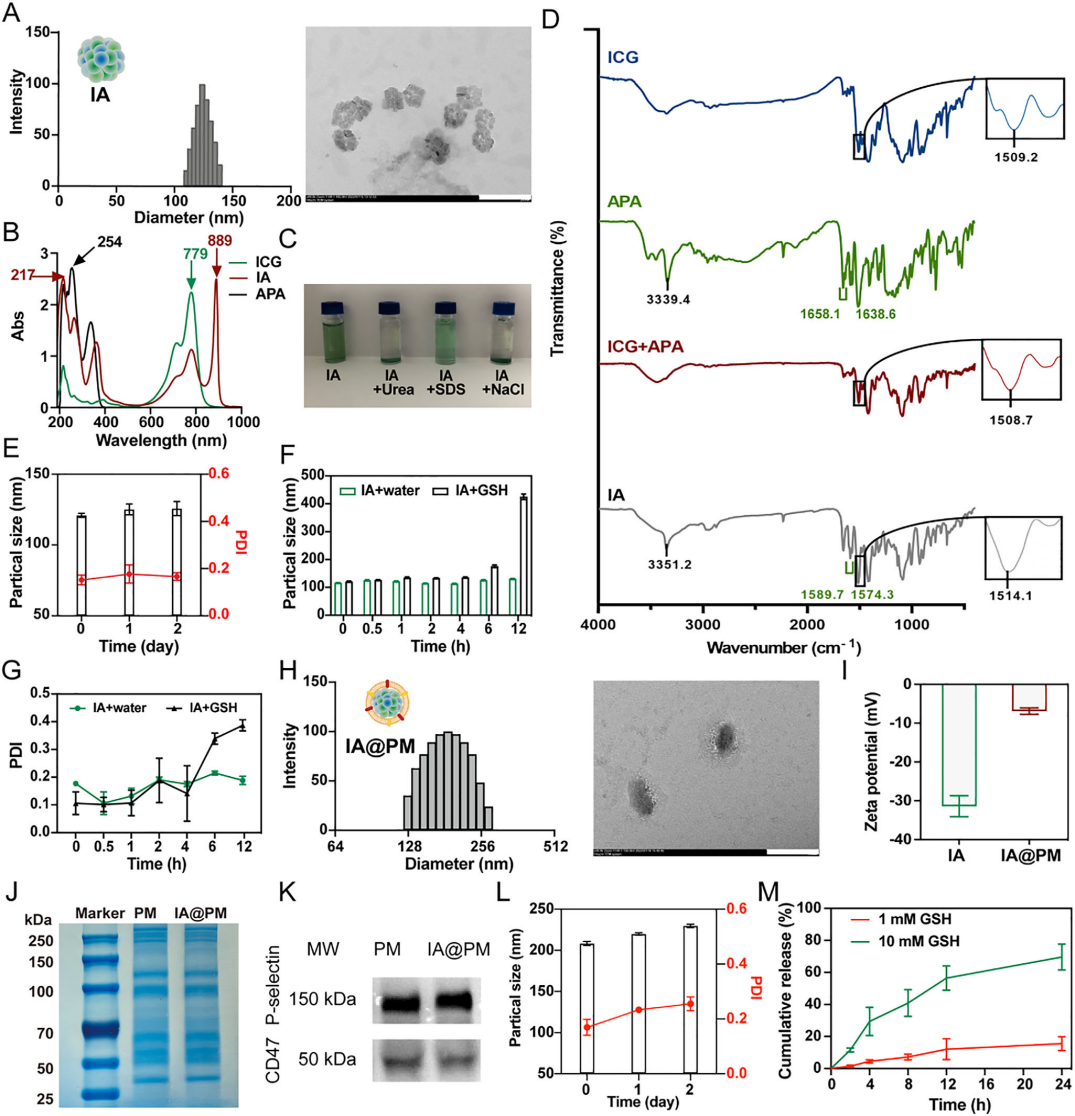
Preparation and characterization of IA and IA@PM. (A) The particle size and TEM image of IA (scale bar: 200 nm). (B) UV–vis spectra of ICG, APA, and IA. (C) IA dissociation by urea, SDS, and NaCl. (D) The FTIR spectrum of ICG, APA, mixture of ICG and APA, and IA. (E) The particle size and PDI changes of IA within 2 days (25°C). The particle size (F) and PDI (G) changes of IA after incubation with water and GSH in 12 h (37°C). (H) The particle size and TEM image of IA@PM (Scale bar: 200 nm). (I) Zeta potential of IA and IA@PM. (J) The SDS‐PAGE analysis of IA@PM. (K) The western bot analysis of IA@PM. (L) The particle size and PDI changes of IA@PM within 2 days (25°C). (M) The drug release of APA in IA@PM with 1 and 10 mM GSH. All error bars indicate S.D. (*n* = 3).

IA was prepared by assembling ICG with APA in aqueous solution (Scheme 1). The particle size and PDI of IA were 129.9 ± 1.7 nm and 0.05 ± 0.01, respectively (Figure S1 and S2, Figure 1A, and Table S1). The TEM image showed that IA was homogeneously distributed and nearly spherical (Figure 1A). Subsequently, the IA assembly was further evaluated with UV–vis analysis (Figure 1B). The characteristic absorption peak of ICG red‐shifted from 779 to 889 nm, and the APA absorption peak blue‐shifted from 254 to 217 nm after IA formation. Meanwhile, the DL of ICG and APA in IA were 13.25% ± 1.30% and 86.75% ± 1.30%, respectively (Table S1). Obvious precipitations were observed immediately after the addition of NaCl (electrostatic force elimination) and urea (hydrogen bonds disruption) (Figure 1C). Besides, after adding SDS (hydrophobic force elimination), both the particle size and UV–vis spectra of IA changed (Figures S3 and S4). These changes suggested that the electrostatic, hydrogen bond, and hydrophobic interaction were the dominant driving forces of IA [22], which was also verified by Fourier transform infrared spectroscopy (FTIR) (Nicolet 5700 FTIR spectrometer) assay. As shown in Figure 1D and Table S2, the *v*_‐NH_ stretching vibration peak of APA at 3339.4 cm^−1^ blue‐shifted to 3351.2 cm^−1^ in IA, suggesting the amino group involved in IA self‐assembly. The characteristic absorption peak of the ICG benzene ring skeleton also varied from 1509.2 to 1514.1 cm^−1^ after IA formation. Meanwhile, the characteristic double peaks of *v*_C = C_ in APA red‐shifted from 1658.1–1638.6 to 1589.7–1574.3 cm^−1^, indicating the π–π interaction within IA. IA nanoparticles exhibited robust stability in dilution (25°C), placement (25°C), and 10% FBS (37°C) conditions (Figure 1E and Figures S5–S7). However, when incubated with 10 mM GSH for 12 h (Figure 1F,G), both the particle size (>400 nm) and PDI (>0.3) of IA notably increased, suggesting its susceptibility to high concentration of GSH.

PM has been utilized as a biomimetic coat to enable nanoparticles to escape from macrophages and recognize tumor cells. It was reported that PM can recognize surface marker proteins CD47 and P‐selectin to facilitate the uptake of the coated nanoparticles to tumor cells [19, 23]. In order to improve the tumor targeting of IA@PM, we coated IA with PM based on the previous reported method. Briefly, PM was assembled onto IA by ultrasonic method to prepare IA@PM. TEM showed a distinct coating shell on IA@PM nanoparticles (Figure 1H). The particle size and zeta potential of IA@PM were 193.0 ± 1.3 nm and −6.9 ± 0.8 mV, respectively. Compared with IA, both the particle size and zeta potential of IA@PM were increased after PM coating. Besides, the PDI of IA@PM was 0.11 ± 0.03 (Figure 1H,I and Figure S2). Moreover, the SDS‐PAGE results indicated that PM and IA@PM exhibited identical protein band profiles (Figure 1J). Notably, IA@PM also displayed the characteristic markers CD47 and P‐selectin associated with PM (Figure 1K). These findings confirmed that nearly all proteins from PM, including CD47 and P‐selectin, were successfully retained on the surface of IA@PM. Similar to IA, IA@PM also possessed good placement (25°C) and 10% FBS (37°C) stability (Figure 1L and Figure S8).

Additionally, as IA was sensitive to the high concentration of GSH, the in vitro drug release behavior of IA@PM was further investigated within different GSH concentrations (Figure 1M). When incubated with 1 mM GSH (phosphate buffer of pH 7.4), only 15.5% of APA was released from IA@PM nanoparticles within 24 h. In contrast, with 10 mM GSH, the corresponding cumulative release of APA reached 71.6%. It was reported that the concentration of GSH in the tumor cells was around 10 mM, whereas the concentration of GSH in normal tissue cells was 0.1–1 mM [24]. In this case, we propose that IA@PM would maintain stability in blood circulation and disassemble in response to the tumor GSH environment.

### 3.2 Tumor Targeting Ability and Selective PDT Effect of IA@PM

**FIGURE 2:**
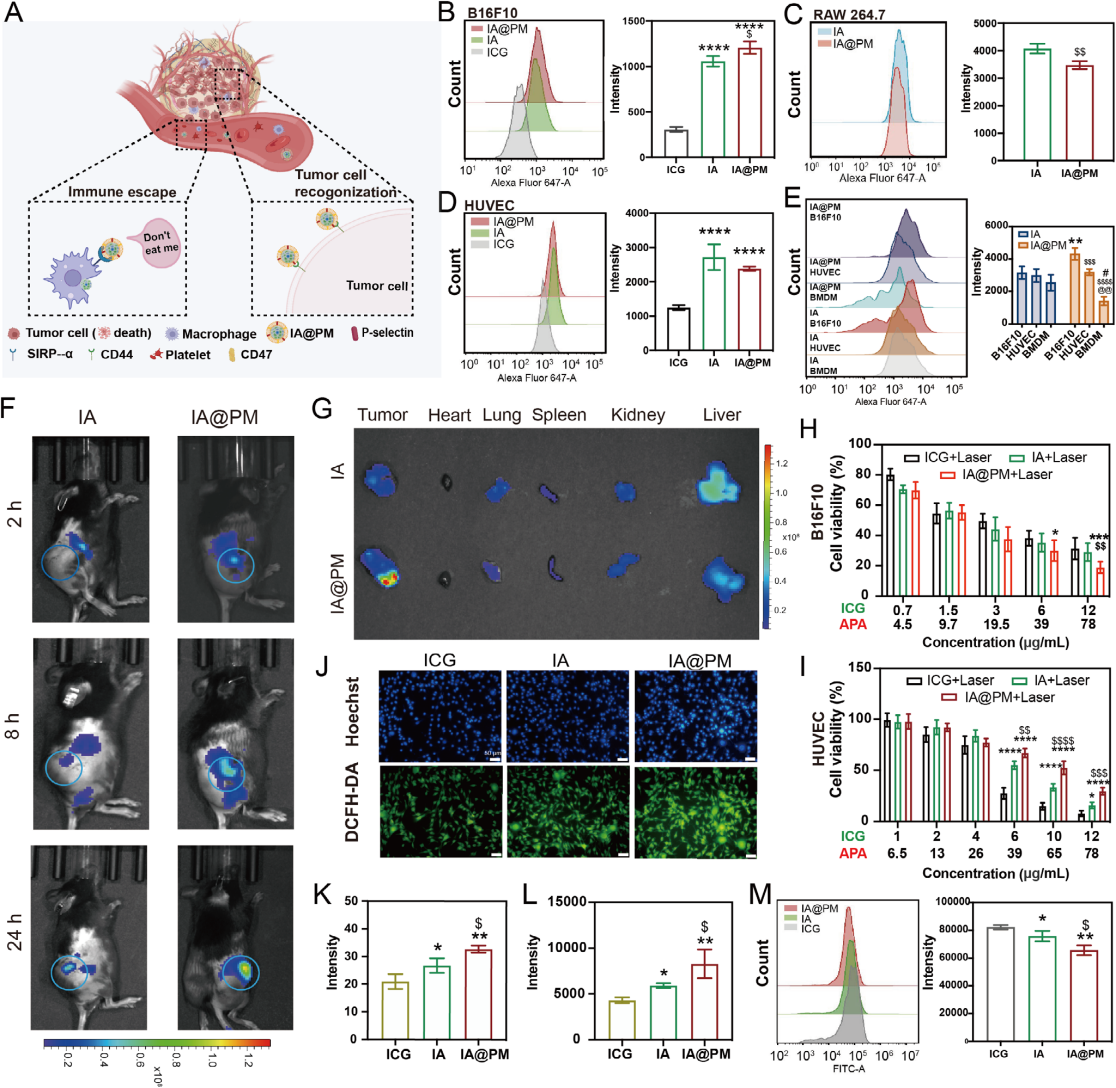
The evaluation of tumor targeting and cytotoxicity of IA@PM. (A) The mechanism of tumor targeting. The fluorescent signal of B16F10 cells (B), RAW 264.7 (C), and HUVEC (D) detected by FCM and quantitative statistics of fluorescence intensity after 12 h incubation with different formulation (*n* = 3). ^****^ *p* < 0.0001 versus ICG, ^$^ *p* < 0.05, and ^$$^ *p* < 0.01 versus IA. (E) FCM analysis of cellular uptake of IA and IA@PM in co‐cultivation of B16F10 cells, HUVEC, and BMDM for 12 h, respectively. ^**^ *p* < 0.01 versus IA B16F10; ^#^ *p* < 0.05 versus IA BMDM; ^$$$^ *p* < 0.001, and ^$$$$^ *p* < 0.0001 versus IA@PM B16F10; ^@@^ *p* < 0.01 versus IA@PM HUVEC. (F) Fluorescence images of B16F10 tumor‐bearing mice at different points of time administrated with IA and IA@PM, respectively. (G) Fluorescence images of major organs obtained from different treated mice at 24 h post‐injection (tumor; heart; lung; spleen; kidney; liver). The cytotoxicity of IA, IA@PM, and ICG under laser against B16F10 cells (H) and HUVEC (I). ^*^ *p* < 0.05, ^***^ *p* < 0.001, and ^****^ *p* < 0.0001 versus ICG + Laser; ^$$^ *p* < 0.01, ^$$$^ *p* < 0.001, and ^$$$$^ *p* < 0.0001 versus IA + Laser (*n* = 6). The fluorescent images (J) and quantitative statistics (K) of DCFH‐DA intensity detected by inverted microscopy in B16F10 cells under 0.8 W cm^−2^ (2 min) laser after 12 h incubation with ICG, IA, and IA@PM (*n* = 3) (scale bar: 100 µm). (L) Quantitative statistics of DCFH‐DA intensity detected by flow cytometry in B16F10 cells under 0.8 W cm^−2^ (2 min) laser after 12 h incubation with ICG, IA, and IA@PM. ^*^ *p* < 0.05 and ^**^ *p* < 0.01 versus ICG; ^$^ *p* < 0.05 versus IA (*n* = 3). (M) The DCFH‐DA intensities and quantitative statistics of fluorescence detected by FCM in HUVEC under laser after 12 h incubation with ICG, IA, and IA@PM (*n* = 3). ^*^ *p* < 0.05, and ^**^ *p* < 0.01 versus ICG; ^$^ *p* < 0.05 versus IA. All error bars indicate S.D.

With the successful incorporation of PM coating onto IA, we propose that IA@PM could accumulate at the tumor sites due to the immune escape and tumor cell selectivity properties of PM [23]. Subsequently, the earlier initiation of VNW, which restores tumor vascular transport capacity, further enhances the intratumoral delivery of IA@PM, thereby creating a self‐amplifying tumor targeting delivery system (Figure 2A). In order to study the internalization of IA@PM in different cell types, IA@PM was incubated with macrophages (RAW 264.7), HUVEC, and B16F10 cells, respectively. As shown in Figure S9, compared to RAW 264.7 cells, IA@PM exhibits a higher propensity for uptake by B16F10 and HUVEC cells. Besides, the intracellular APA concentration in the IA@PM group was significantly higher in B16F10 cells and lower in RAW 264.7 compared with IA group at 8 and 12 h after incubation (*p* < 0.01–0.05) (Figure S10), which was further verified by FCM (*p* < 0.01–0.05) (Figure 2B,C). In addition, PM modification did not affect the uptake efficiency of IA by HUVEC (Figure 2D). These characteristics may facilitate the tumor targeting of IA@PM during the systemic circulation following drug administration. Concurrently, to simulate TME in vivo, a co‐culture model of B16F10, HUVEC, and BMDM was established. Compared with macrophages, IA@PM exhibited a higher propensity for uptake by tumor cells, while there was no significant difference in the uptake of IA among the three cell lines (Figure 2E). These results demonstrated that IA@PM possessed enhanced tumor cell targeting ability, which is attributed to the PM coating.

The biodistribution of IA@PM in B16F10 tumor‐bearing mice was also investigated through in vivo image technology. The fluorescence of ICG appeared at the tumor site at 2 h after *i.v*. and continued to intensify within 24 h in the IA@PM group (Figure 2F). In contrast, in the IA group, fluorescence was not detected at the tumor site until 8 h post‐injection and kept weaker than IA@PM from 8 to 24 h. Then, the tumor‐bearing mice were sacrificed at 24 h post‐injection. The fluorescence intensity detected from the *ex*‐tissues also illustrated the superior tumor targeting ability of IA@PM compared with IA (*p* < 0.01) (Figure 2G and Figure S11).

Next, we further performed MTT analysis on B16F10 cells and HUVEC. None of the formulations displayed obvious cytotoxicity against either cell type in the absence of laser irradiation, suggesting good biocompatibility (Figures S12 and S13). Upon laser irradiation, IA@PM (IC_50_ 2.73 µg mL^−1^) exhibited better B16F10 cell inhibition efficacy than IA (IC_50_ 3.13 µg mL^−1^) and ICG (IC_50_ 3.63 µg mL^−1^). However, this trend was completely reversed in HUVECs, where the cytotoxicity of IA@PM was significantly lower than that of IA under laser treatment (*p* < 0.0001–0.01) (Figure 2H,I). These observed results can be attributed to variability in the ability of IA@PM to generate ROS which are known for their potent cytotoxic effects, across different cell lines. As shown in Figure 2J–L and Figure S14, compared with IA, IA@PM produced significantly higher ROS in B16F10 (*p* < 0.05). In contrast, IA@PM produced remarkably lower ROS in HUVEC compared with IA (*p* < 0.05) (Figure 2M and Figure S15). In fact, the reduced level of ROS generation by IA@PM in HUVEC is likely to enhance its ability to repair tumor vasculature in vivo. Excessive ROS can induce a highly potent cytotoxic effect on VECs, leading to excessive pruning of the vasculature and finally leading to an anti‐angiogenic effect rather than a VNT effect.

### 3.3 IA@PM Accelerating VNW Initiation

To investigate the anti‐angiogenic effect of IA and IA@PM, HUVEC migration was assessed (Figure S16). After co‐incubated with IA or IA@PM, the migration rates of HUVEC (12.4% ± 4.1% and 13.2% ± 6.8%) were remarkably lower than that of the control group (30.1% ± 3.3%) (*p* < 0.01), which was attributed to the VECs migration inhibition effects of APA [25]. To investigate the role of ROS in inhibiting VECs migration, a treatment group was established using H_2_O_2_, a type of ROS. After incubation with 100 µM H_2_O_2_ (simulating the TME), HUVECs exhibited a migration rate similar to that of the control group (Figure S17). When the H_2_O_2_ concentration was increased to 200 µM (simulating exogenous ROS), the migration rate of HUVECs decreased to 9.3% ± 1.4%, suggesting that ROS have the potential to inhibit VECs migration. Consequently, ICG + Laser treatment also effectively inhibited HUVEC migration (4.3% ± 1.3%). Furthermore, compared to IA@PM without ROS production, IA@PM + Laser exhibited superior inhibition of HUVEC migration (*p* < 0.01). This finding supports the effectiveness of PDT in enhancing the VNT efficacy of anti‐VEGF monotherapy mediated by APA through the inhibition of VECs migration.

**FIGURE 3:**
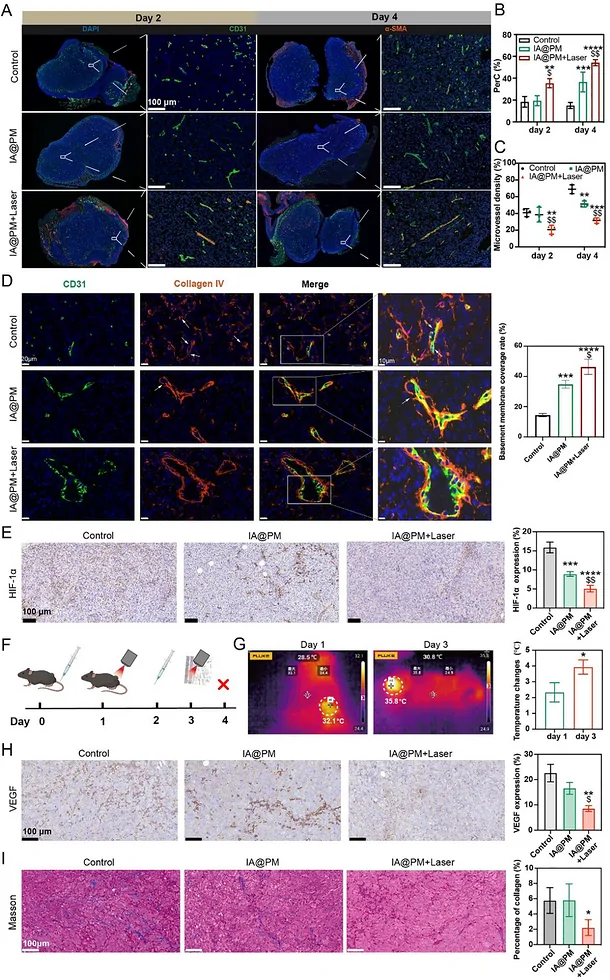
The evaluation of VNW initiation. (A) Images of tumor microvessels in B16F10 tumor‐bearing mice treated with different formulations on day 2 and day 4. Endothelial cells and pericytes were stained with CD31 (green) and α‐SMA (red) (scale bar: 100 µm); (B) Quantification statistics of PerC for each group; (C) Quantification statistics of MVD for each group. (D) Images and quantification statistics of BM coverage rate for each group on day 2, respectively. Endothelial cells and BM were stained with CD31 (green) and Collagen IV (red). White arrows showed the leaky membrane, and purple arrows showed the empty sleeves of membrane (scale bar: 20 µm, chosen pictures scale bar: 10 µm). (E) Immunohistochemistry staining images and quantification of HIF‐1α (brown) (scale bar: 100 µm) treated with different administrations on day 2. (F) Schematic plans for the administration of VNT in melanoma model (saline, IA@PM, and IA@PM + Laser). (G) The temperature changes of tumor site after laser irradiation on day 1 and day 3. (H) Immunohistochemistry staining images and quantification of VEGF (brown) (scale bar: 100 µm) treated with different administrations on day 2. (I) Images and quantification statistics of Masson staining (collagen: blue) in tumor tissues on day 2 (scale bar: 100 µm). ^*^ *p* < 0.05, ^**^ *p* <0.01, ^***^ *p* < 0.001, and ^****^ *p* < 0.0001 versus control; ^$^ *p* < 0.05, and ^$$^ *p* < 0.01 versus IA@PM. All error bars indicate S.D. (*n* = 3).

Next, the melanoma tumor mouse model was employed to explore the VNW initiation effect of IA@PM in vivo, the doses and time intervals of administrations were determined by the optimal VNT efficacy according to the previous study [7]. We collected tumor samples on day 2 and day 4 post‐treatment to assess the degree of VNT. The pericyte coverage (PerC) rate was used as the indicator of vascular maturation [26]. The corresponding tumor slices were double stained with CD31 (VECs, green) and α‐SMA (pericytes, red) maker to evaluate the PerC ratio of tumor vessels. On day 2, compared with the IA@PM group, the PerC in the IA@PM + Laser group (35.4% ± 4.1%) was significantly higher than that in the control group (*p* < 0.01). This indicated that IA@PM initiated vascular normalization and produced a superior tumor vascular repair effect by day 2 (Figure 3A,B). Moreover, we also observed that the initiation of VNW in the IA@PM group occurred on day 4, which was significantly later than the IA@PM + Laser group. These results further emphasized that moderate ROS generation‐associated PDT treatment can effectively promote VNW initiation. More than this, on day 4, IA@PM + Laser exhibited higher PerC rate (54.2% ± 2.8%) than IA@PM based on anti‐VEGF monotherapy (*p* < 0.01), suggesting that IA@PM + Laser could not only accelerate VNW initiation but also enhance VNT effects. Furthermore, compared with twisted blood vessels in the control group and IA@PM group, the IA@PM + Laser group exhibited relatively regular vessels and significantly decreased microvessel density (MVD) on both days 2 and 4 (*p* < 0.001–0.01) (Figure 3C). These results suggested that IA@PM accelerated the initiation of the VNW (on day 2) by efficiently repairing immature vessels and pruning aberrant tumor vasculature.

Additionally, the state of the basement membrane (BM) is a critical factor influencing the VNT efficacy. Accumulation of BM leads to abnormal vessel curvature, whereas the absence of BM results in vessel leakage. Moreover, the presence of excessive empty BM sleeves indicates a deterioration in vascular function [27, 28]. Accordingly, we investigated the BM coverage ratio to further assess the repair effects of VNT on day 2 post‐treatment (Figure 3D). Both the control and IA@PM groups presented BM absence (white arrows), accompanied by BM accumulation and distortion. Especially in the control group, there was a mass of empty BM sleeves and irregular vessel structure. In contrast, tumor vessel system was improved in all treatment groups, with more BM distribution and more complete BM coverage (46.4% ± 5.0%) after IA@PM + Laser treatment (*p* < 0.0001–0.05). These results suggested that with the help of controlled PDT, IA@PM + Laser treatment offered the additional benefit of improving the status of the tumor vascular BM. In addition, considering the hypoxic tumor environment can lead to the suboptimal oxygen‐dependent PDT effects, we further investigated vessel repair effects of IA@PM with the index hypoxia inducible factor α (HIF‐1α) [29, 30]. The broader expression of HIF‐1α (brown dots) was observed in the control group (17.5% ± 3.5%) on day 2, and IA@PM (8.6% ± 1.7%) and IA@PM + Laser (5.0% ± 2.2%) effectively downregulated HIF‐1α expression (*p* < 0.0001–0.001) (Figure 3E). This result suggested that IA@PM + Laser treatment could also mitigate the unwanted aggravated tumor hypoxia followed the conventional PDT. Interestingly, we also found that upon under constant laser irradiation conditions and administered dose, the temperature of tumor site increased after the second laser irradiation compared to the first laser irradiation (*p* < 0.05) (Figure 3F,G), which implied greater tumor targeting of IA@PM might be occurred at the second treatment. This effect might be attributed to the rapid repair of tumor vasculature by IA@PM following the initial treatment, which could further increase drug enrichment in the tumor after the second administration.

Encouraged by these results, we aim to elucidate the mechanism of how controllable PDT facilitated VNW initiation and enhances VNT. To begin, we investigated alterations in the levels of the classical pro‐angiogenic factor VEGF within tumors. All treatment groups exhibited a significantly downregulated VEGF level than the control group (*p* < 0.01) on day 2 (Figure 3H). Especially, when combined with PDT, IA@PM + Laser (8.6% ± 1.2%) resulted in significantly greater downregulation of VEGF compared to IA@PM (16.6% ± 3.4%) (*p* < 0.05). This suggested that PDT further accelerated the anti/pro‐angiogenic balance, thereby promoting VNW initiation. Except for VEGF, tumor angiogenesis was also highly associated with the complicated ECM environment. On the one hand, the excessive tumor ECM led to increased solid stress, which aggravated vessel leakage and abnormal vessel structure. On the other hand, the tumor‐associated fibroblast was proven to secrete pro‐angiogenetic factors, promoting tumor angiogenesis [31]. Accordingly, Masson staining, a classical method for detecting major fiber components in ECM, was conducted to test the ECM changes after different treatment. As shown in Figure 3I, the IA@PM + Laser group exhibited the lowest Masson staining positive ratio (2.3% ± 0.4%) on day 2. On day 4, the difference between IA@PM + Laser and other groups increased (*p* < 0.0001), and there was no noticeable increase in the Masson expression area compared with that of day 2 (Figure S18). These results suggested that with the help of controllable PDT, IA@PM could effectively decrease the tumor ECM dense. Leveraging these ECM remodeling effects of IA@PM + Laser, ECM‐induced vascular leakage, vascular anomalies, and pro‐angiogenic effects can be effectively suppressed. Taken together, IA@PM + Laser‐mediated moderate PDT treatment could promote accelerated VNW initiation and VNT potentiation by downregulating tumor VEGF levels and remodeling the ECM.

**FIGURE 4:**
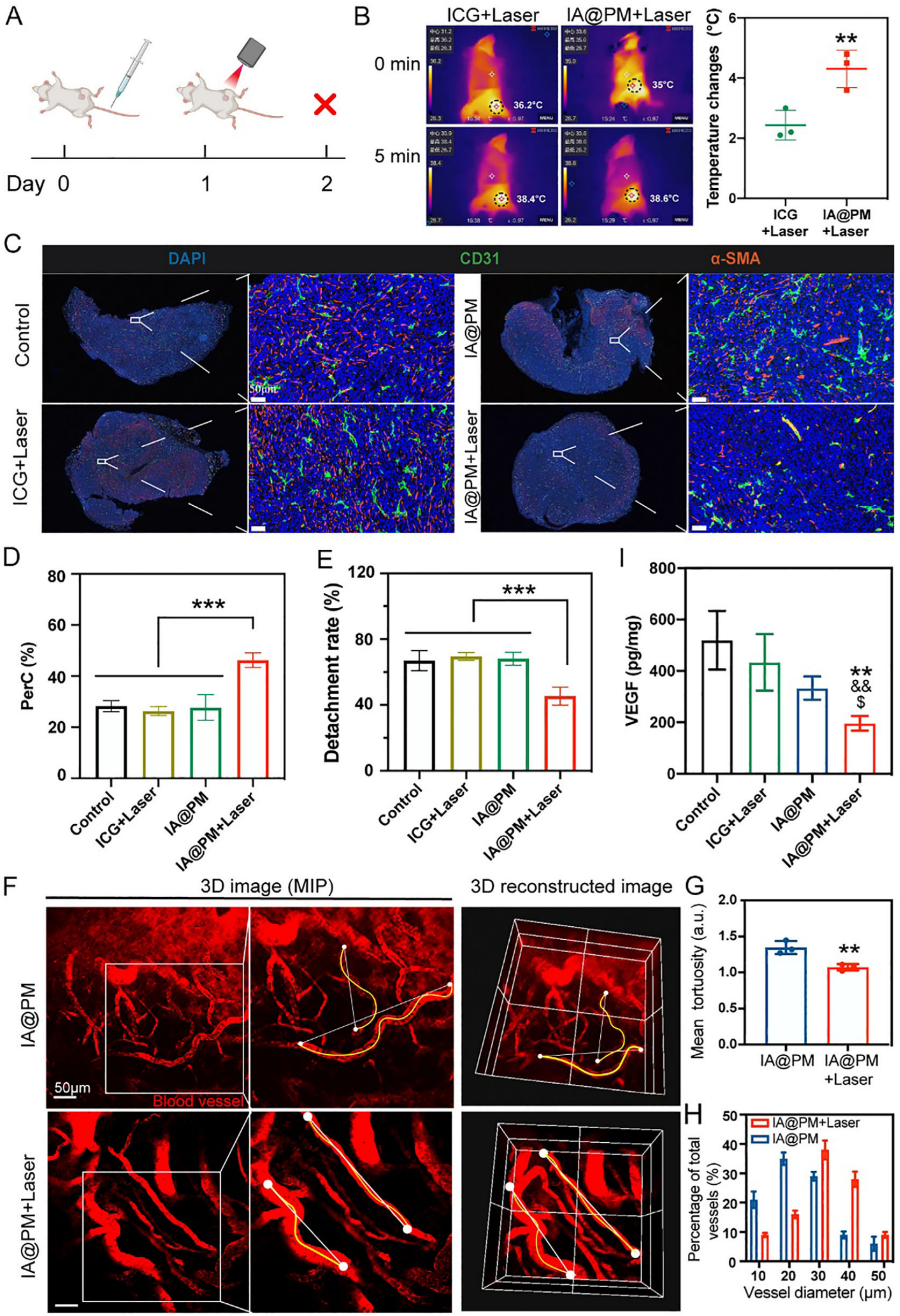
The evaluation of VNW initiation. (A) Schematic plans for the administration of VNT in orthotopic breast cancer (saline, ICG + Laser, IA@PM, and IA@PM + Laser). (B) The temperature of tumor after laser irradiation on day 1. ^**^ *p* < 0.01 versus ICG + Laser. VNT effects of IA@PM: (C) The images of tumor vessels in 4T1 tumor‐bearing mice treated with different formulations on day 2, respectively. Endothelial cells and pericytes were stained with CD31 (green) and α‐SMA (red) (Scale bar: 50 µm); (D) Quantification statistics of PerC for each group; (E) Quantification statistics of detachment rate for each group. ^***^ *p* < 0.001 versus IA@PM + Laser. (F) Two‐photon 3D image of tumor treated with IA@PM and IA@PM + Laser (scale bar: 50 µm). The corresponding vessel tortuosity (G) and vessel diameter distribution (H) of blood vessels. ^**^ *p* < 0.01 versus IA@PM. (I) The VEGF level of different groups detected by the ELISA kit. ^**^ *p* < 0.01 versus control; ^&&^ *p* < 0.01 versus ICG + Laser; ^$^ *p* < 0.05 versus IA@PM. All error bars indicate S.D. (*n* = 3).

In addition, breast cancer often exhibits vascular invasion, and its therapeutic efficacy is frequently constrained by the tumor abnormal vascular network. Encouraged by the superior therapeutic effect of IA@PM + Laser for melanoma, we further investigated its therapeutic efficacy in treating breast cancer using a 4T1 breast tumor mouse model (Figure 4A). Consistent with the phenomenon of IA@PM used for treatment in a mouse model of melanoma, the temperature of tumor site of IA@PM + Laser group was higher than that of the ICG + Laser (*p* < 0.01) (Figure 4B). Besides, we also assessed the PerC ratio of different treated groups (Figure 4C,D). On day 2, there was a significantly higher PerC in IA@PM + Laser (44.2% ± 2.9%) than that in other groups (*p* < 0.001). Then, considering that the abnormal vessels of 4T1 tumors typically exhibit a high rate of pericyte detachment, the detachment rate was subsequently calculated. On day 2, compared to other groups (>65%), the IA@PM + Laser group exhibited VECs with closely covered pericytes and lowest detached pericytes (40%), suggesting the improved integrity of vessels (*p* < 0.001) (Figure 4E). Furthermore, as three‐dimensional (3D) imaging‐based analysis offers a clear and unbiased means to quantify the tortuosity of blood vessels, we conducted the two‐photo 3D in vivo imaging of the mice treated with IA@PM and IA@PM + Laser. The tortuosity of the blood vessels in the IA@PM + Laser treated group was significantly lower than that of the IA@PM treated group (*p* < 0.01) (Figure 4F,G). Meanwhile, compared to the IA@PM group, we observed that the vessel diameter was notably increased in the IA@PM + Laser treatment group (Figure 4H), which could further enhance blood perfusion to the tumor site. The vessel distribution in the IA@PM + laser treatment group was between 30 and 40 µm, which effectively reduced the occupation of microvessels (<20 µm). Moreover, VEGF levels in the tumors following different treatments were also assessed (Figure 4I). There were no significant differences in VEGF level among ICG + Laser, IA@PM, and the control groups. In contrast, IA@PM + Laser reduced the VEGF level to 196.3 ± 28.5 pg mg^−1^, which was also significantly lower than that of the IA@PM group (*p* <0.05). These results indicated that IA@PM + Laser could effectively mediate VNT potentiation in breast cancer, suggesting that IA@PM + Laser might have the potential to target different vascular‐rich tumors.

### 3.4 The Antitumor Effect Evaluation and VNT Promotion Effects of IA@PM

**FIGURE 5:**
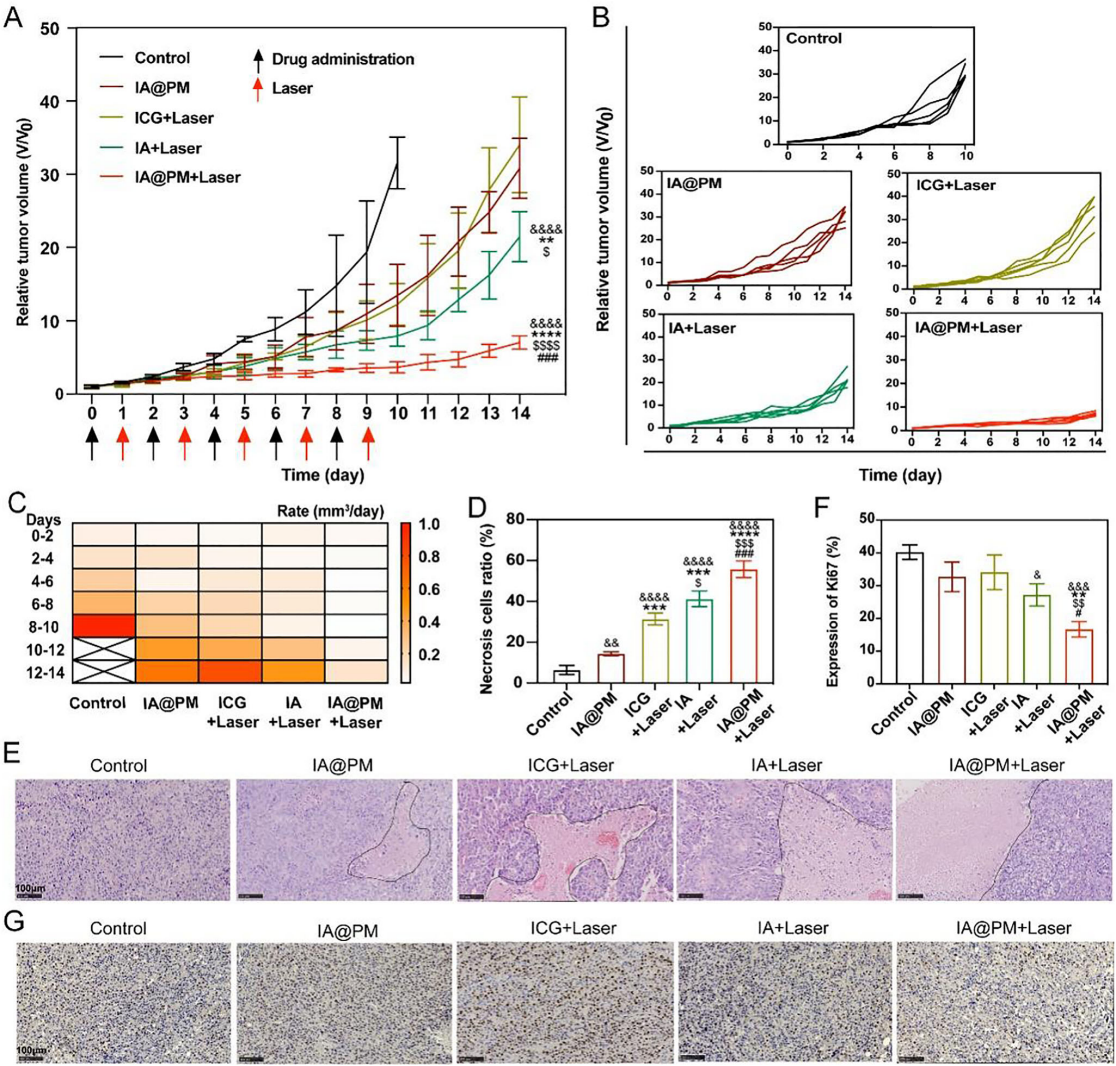
The antitumor effect evaluation of IA@PM. (A) The relative tumor growth curves after treated with different administrations: Control (saline); IA@PM; ICG + Laser; IA + Laser; IA@PM + Laser. The black arrow means vascular normalization drug (*i.v*.), the red arrow means laser. (B) Individual relative growth of tumor in different treatment groups. (C) The relative tumor progression of mice treated with different administrations. The quantitative statistics of apoptotic cells (D) and H&E staining images (E) of tumor treated with different treatments. The black circles indicate the apoptotic area (scale bar: 100 µm). The quantitative statistics of Ki67 expression (F) and Ki67 staining images (G) of tumor treated with different treatments (scale bar: 100 µm). ^&^ *p* < 0.05, ^&&^ *p* < 0.01, ^&&&^ *p* < 0.001, and ^&&&&^ *p* < 0.0001 versus control; ^**^ *p* < 0.01, ^***^ *p* < 0.001, and ^****^ *p* < 0.0001 versus IA@PM; ^$^ *p* < 0.05, ^$$^ *p* < 0.01, ^$$$^ *p* < 0.001, and ^$$$$^ *p* < 0.0001 versus ICG + Laser; ^#^ *p* < 0.05, and ^###^ *p* < 0.001 versus IA + Laser. All error bars indicate S.D. (*n* = 5).

The antitumor effect evaluation of IA@PM was evaluated in the melanoma model. As displayed in Figure 5A,B and Table S3, compared with the control group, all four treatment groups inhibited tumor growth to varying degrees. Although the PDT‐mediated treatment showed a slower tumor growth rate than mono‐VNT therapy, both ICG + Laser and IA + Laser failed to inhibit tumor growth after treatment withdrawal (days 10–12) (Figure 5C). In contrast, IA@PM + Laser was found to be most effective in inhibiting tumor growth, achieving sustained antitumor effects and a significantly enhanced tumor suppression rate. Furthermore, the tumors were collected for H&E staining (Figure 5D,E). The necrosis rate in the IA@PM + Laser group was 54.5% ± 3.6%, which was remarkably higher than that of other groups (*p* < 0.0001–0.001). In addition, we also tested the Ki67 levels of the tumors after treatment, which is a well‐established biomarker commonly used in clinical practice to evaluate the efficacy of antitumor therapy. As shown in Figure 5F,G, the IA@PM + Laser group displayed the lowest proportion of Ki67‐positive cells than other groups. Collectively, these results indicated that IA@PM combined with PDT showed the excellent anti‐tumor effect in the melanoma model. We propose that the superior antitumor efficacy of IA@PM + Laser is attributable to a dynamic self‐amplifying mechanism in which ROS play a crucial and multifaceted role. Initially, IA@PM + Laser induced early‐onset vascular normalization (on day 2 post‐treatment) via controlled ROS‐mediated mechanisms. As treatment progressed, this vascular normalization facilitated the enrichment of IA@PM at the tumor site during later treatments. Benefiting from this improved drug delivery to the tumor, by the fourth to fifth treatments, this drug enrichment enabled a photothermal therapy (PTT) effect even at ICG doses insufficient to produce a therapeutic PTT effect, as evidenced by temperature changes comparable to those observed in conventional PTT treatments (Figure S19). This gradual approach allowed for vascular normalization in the early stages while transitioning to tumor suppression through enhanced ROS production mediated by PDT and PTT in the later stages of treatment. This strategy also finely tuned progression prevented intense PTT that could impede treatment in the early stages while providing a gentle synergistic boost to enhance overall antitumor efficacy.

As malignant melanoma is one of the most aggressive tumor with high metastasis [32], the anti‐metastasis efficacy of IA@PM was also investigated. The wound healing analysis of B16F10 cells was conducted primarily to validate the therapeutic effects on cell migration. Figure S20 indicated that all treatment groups effectively inhibited B16F10 cell migration (*p* < 0.0001). IA@PM + Laser remarkably inhibited melanoma cell migration (4.4% ± 1.6%), exhibiting its potent anti‐metastasis effect. Furthermore, the IA@PM + Laser group exhibited significantly fewer metastatic nodules compared to the other groups (Figure S21). These findings underscore the exceptional anti‐metastatic efficacy of the combination therapy in inhibiting tumor proliferation and progression.

**FIGURE 6:**
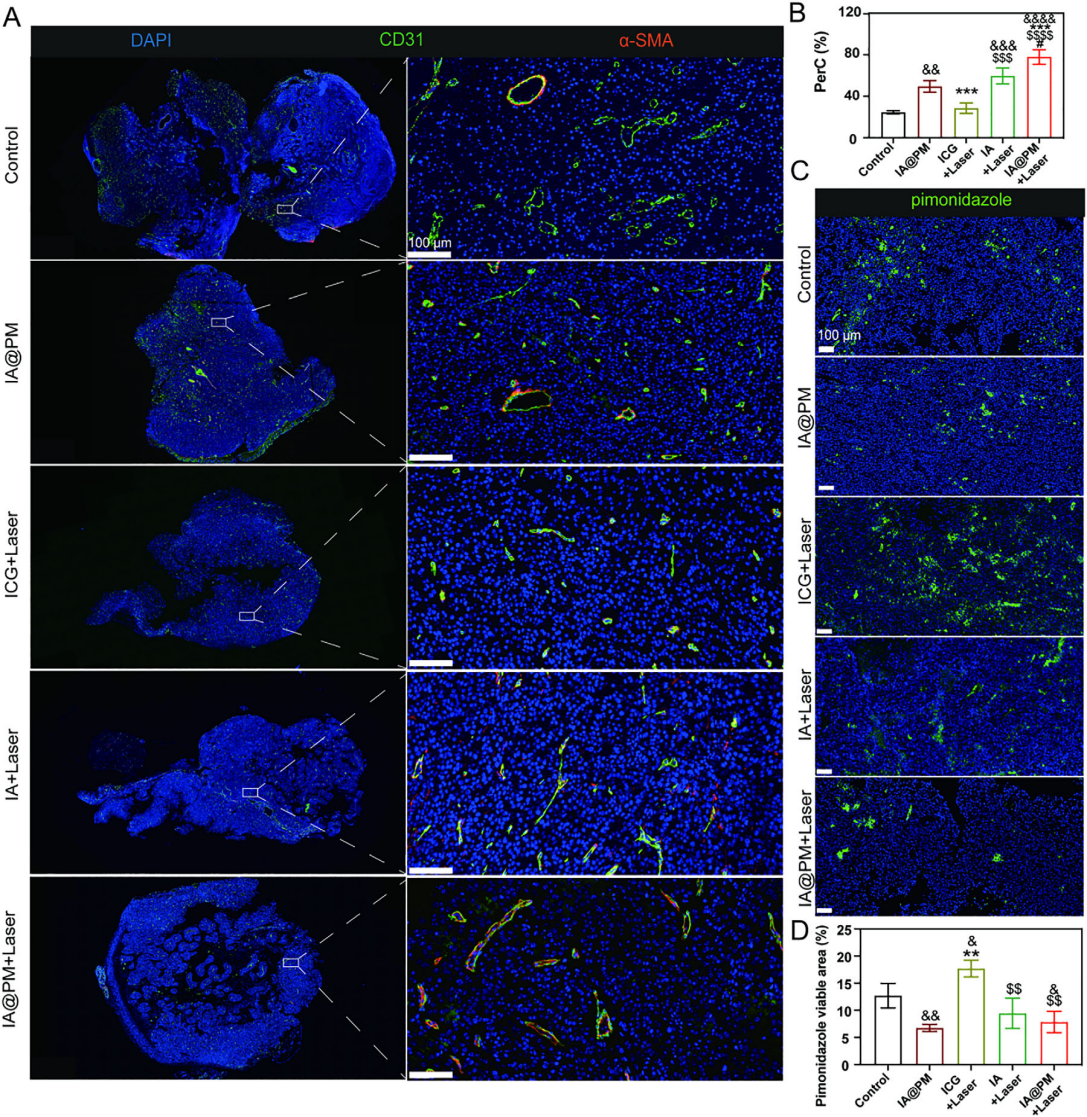
The VNT effect evaluation of IA@PM. (A) The images of tumor vessels in B16F10 tumor bearing mice treated with saline, IA@PM, ICG + Laser, IA + Laser, and IA@PM + Laser. Endothelial cells, nuclear and pericytes were stained with CD31 (green), DAPI (blue), and α‐SMA (red), respectively (scale bar: 100 µm), on day 8 after *i.v*. (B) Quantification of PerC for each group. The images (C) and quantification of pimonidazole area (D) on day 8 after *i.v*. Nuclear and pimonidazole were presented as blue (DAPI) and green, respectively (scale bar: 100 µm). ^&^ *p* < 0.05, ^&&^ *p* < 0.01, ^&&&^ *p* < 0.001, and ^&&&&^ *p* < 0.0001 versus control; ^**^ *p* < 0.01 and ^***^ *p* < 0.001 versus IA@PM; ^$$^ *p* < 0.01, ^$$$^ *p* < 0.001, and ^$$$$^ *p* < 0.0001 versus ICG + Laser; ^#^ *p* <0.05 versus IA + Laser. All error bars indicate S.D. (*n* = 3).

Next, having demonstrated that IA@PM + Laser accelerated initiation of the VNW to day 2, we sought to determine if these enhanced VNT effects were sustained and benefit the antitumor effect of IA@PM + Laser treatment. Given that APA demonstrated the most significant VNT effect on day 8 in previous research [7], we proceeded to investigate the VNT effect of IA@PM + Laser on the day 8. The PerC markedly increased to 77.9% ± 7.0% in the IA@PM + Laser group, compared with 59.6% ± 7.6% in the IA + Laser group and 49.9% ± 5.6% in the IA@PM group (*p* < 0.001–0.05) (Figure 6A,B). In sharp contrast, the PerC was only 28.6% ± 5.0% in the ICG + Laser group and 24.6% ± 1.5% in the control group (*p* < 0.001), which indicated that IA@PM + Laser improved the effects and extended the VNW on the basis of accelerating the VNW initiation. However, repeated PDT was reported to aggravate intratumor hypoxia, upregulating HIF‐1α and subsequent elevation of VEGF levels [33, 34]. Accordingly, ICG + Laser resulted in a significant exacerbation of tumor hypoxia compared to the control group (*p* < 0.05) (Figure 6C,D). Meanwhile, though the IA + Laser group did not exhibit a significant difference compared to the control group, it did maintain less pimonidazole (+) area than the ICG + Laser group. In contrast, the IA@PM + Laser group exhibited a substantial alleviation of the hypoxic conditions than the control group (*p* < 0.05). Based on these results, we concluded that IA@PM + Laser treatment facilitated the mutual promotion of VNT and PDT for vascular normalization and antitumor effects, attributing to the PDT‐induced VNT promotion and breaking the negative feedback loop of PDT with the assistance of VNT.

### 3.5 Biosafety Evaluation of IA@PM

To investigate the safety profiles of IA@PM, a hemolytic assay was conducted. The hemolysis rates for ICG, IA, and IA@PM ranged from 1% to 5%, indicating no significant hemolytic effect, particularly for IA@PM (Figure S22). No significant body weight loss was observed in any of the groups on day 14 compared to day 0 (Figure S23). Additionally, H&E images of the main organs revealed no damage in any of the groups (Figure S24). Liver function indices, including ALT and AST, were evaluated, and no significant differences were observed among the five groups (Figure S25). These results collectively indicate a favorable safety profile for IA@PM in vivo.

## 4 Conclusion

In this study, we constructed the self‐assembly nanoparticles IA, which were further coated with PM to form biomimetic nanoparticles IA@PM. Under laser irradiation, the IA@PM was verified to kill tumor cells effectively while avoiding excessive pruning vessels, which facilitated the rapid regulation of pro‐angiogenesis factors and modulated the tumor ECM through a self‐amplifying tumor targeting method. After being treated with the IA@PM + Laser treatment, both vessel structure and function were normalized on day 2 (monotherapy of IA@PM on day 4) and remained optimal on day 8. Furthermore, VNT effects eliminated the negative feedback of repeated PDT and improved the tumor hypoxia, facilitating the mutual promotion of PDT and VNT, thereby constructing the virtuous therapeutic cycle. The IA@PM + Laser treatment also effectively inhibited tumor proliferation and metastasis by regulating the tumor vessels and ECM systems. Collectively, the IA@PM + Laser treatment provided a novel method to break the clinical bottleneck of VNT application. Previously, ROS was primarily acknowledged for directly killing tumors and adversely affecting blood vessels. This study sheds light on its potential as an adjunctive agent in VNT, underscoring its ability to facilitate vascular normalization and improve therapeutic outcomes. Overall, by addressing the critical challenge of delayed VNW initiation, this work offers new insights into improving the timeliness and efficacy of VNT, potentially benefiting patients with aggressive solid tumors.

## Author Contributions

**Yufei Liu**: methodology, data curation, writing – original draft, investigation. **Changheng Xie**: formal analysis, data curation, investigation. **Yanfeng Huang**: investigation. **Ting Wang**: investigation. **Shi Du**: supervision, writing – review and editing. **Hui Xiong**: supervision, writing – review and editing, investigation. **Jing Yao**: supervision, conceptualization, funding acquisition.

## Funding

The authors have nothing to report.

## Conflicts of Interest

We declare that we have no known competing financial interests or personal relationships that could have appeared to influence the work reported in this paper.

## Supporting information




**Supporting File 1**: exp270180‐sup‐0001‐SuppMat.docx.

## Data Availability

Data will be made available on request.
