# Supplementary material for: Photodynamic Biomimetic Nanoparticles Accelerate Tumor Vascular Normalization Initiation
Source: Exploration (Beijing). 2026 Jun 5:20240333. Online ahead of print. doi: 10.1002/EXP.20240333 (PMC13394804; doi:10.1002/EXP.20240333)
Supplement: Supplementary file 1 — Supporting File 1: exp270180‐sup‐0001‐SuppMat.docx. [file EXP2-9999-0-s001.docx]

Supporting Information

Photodynamic Biomimetic Nanoparticles Accelerate Tumor Vascular Normalization Initiation

*Yufei Liu ^a^, Changheng Xie ^a^, Yanfeng Huang ^a^, Ting Wang ^a^, Shi Du ^b, c, *^, Hui Xiong ^a,*^, Jing Yao ^a,*^*.

^a^Jiangsu Key Laboratory of Druggability of Biopharmaceuticals, Department of Pharmaceutics, China Pharmaceutical University, 639 Longmian Avenue, Nanjing 211198, P. R. China.

^b^International College of Pharmaceutical Innovation, Soochow University, Suzhou, 215222, P. R. China.

^c^Department of Biomedical Informatics, College of Medicine, The Ohio State University, Columbus, OH 43210, USA.

^*^Corresponding authors: E-mail: yaojing@cpu.edu.cn (J Yao), 1620174410@cpu.edu.cn (H Xiong), and dushi@suda.edu.cn (S Du).


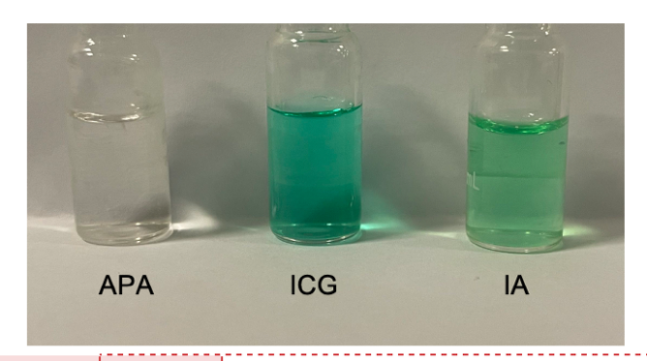


**Figure S1.** The picture of APA, ICG, and IA solution.

**Figure S2.** The particle size and PDI of IA and IA@PM, respectively. All error bars indicate S.D. (n = 3).


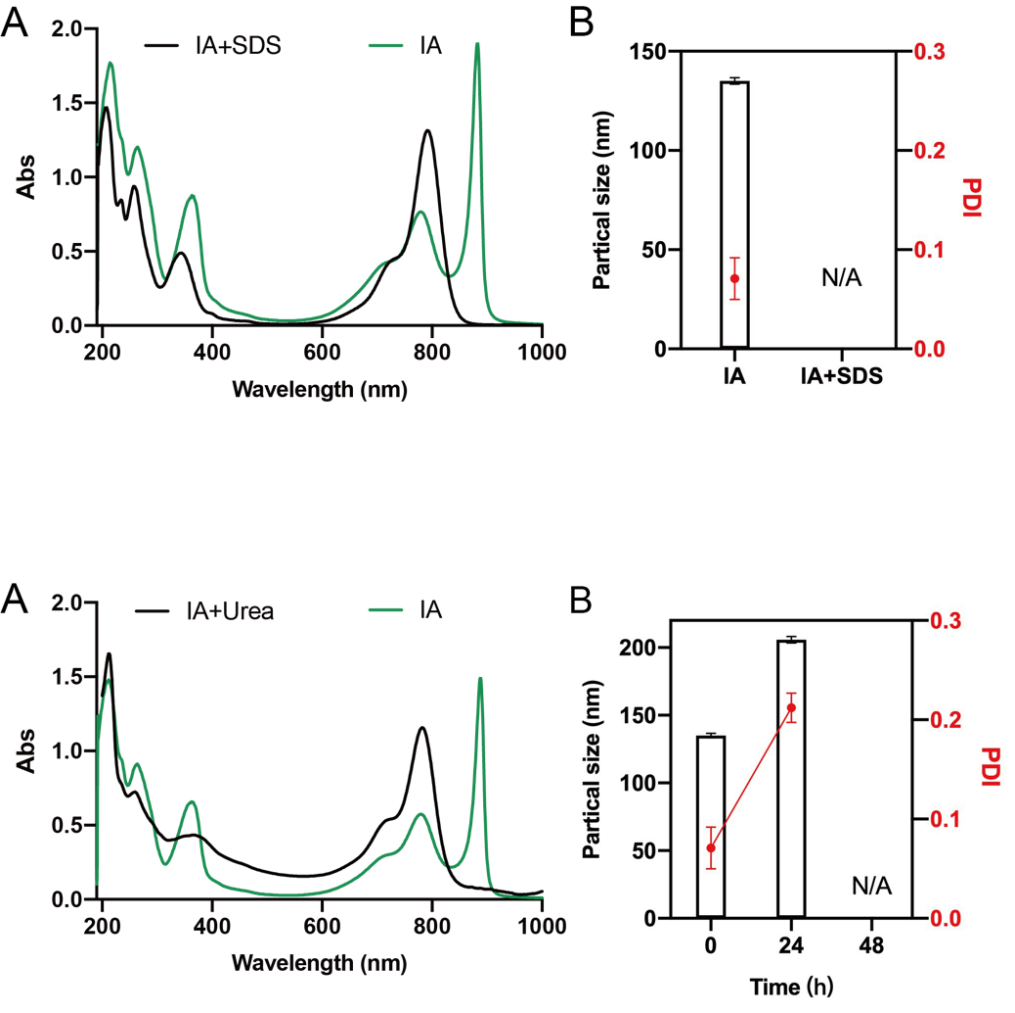


**Figure S3.** (A) UV-vis spectra of IA and IA+Urea. (B) The particle size and PDI changes of IA+Urea within 48 h. All error bars indicate S.D. (n = 3).


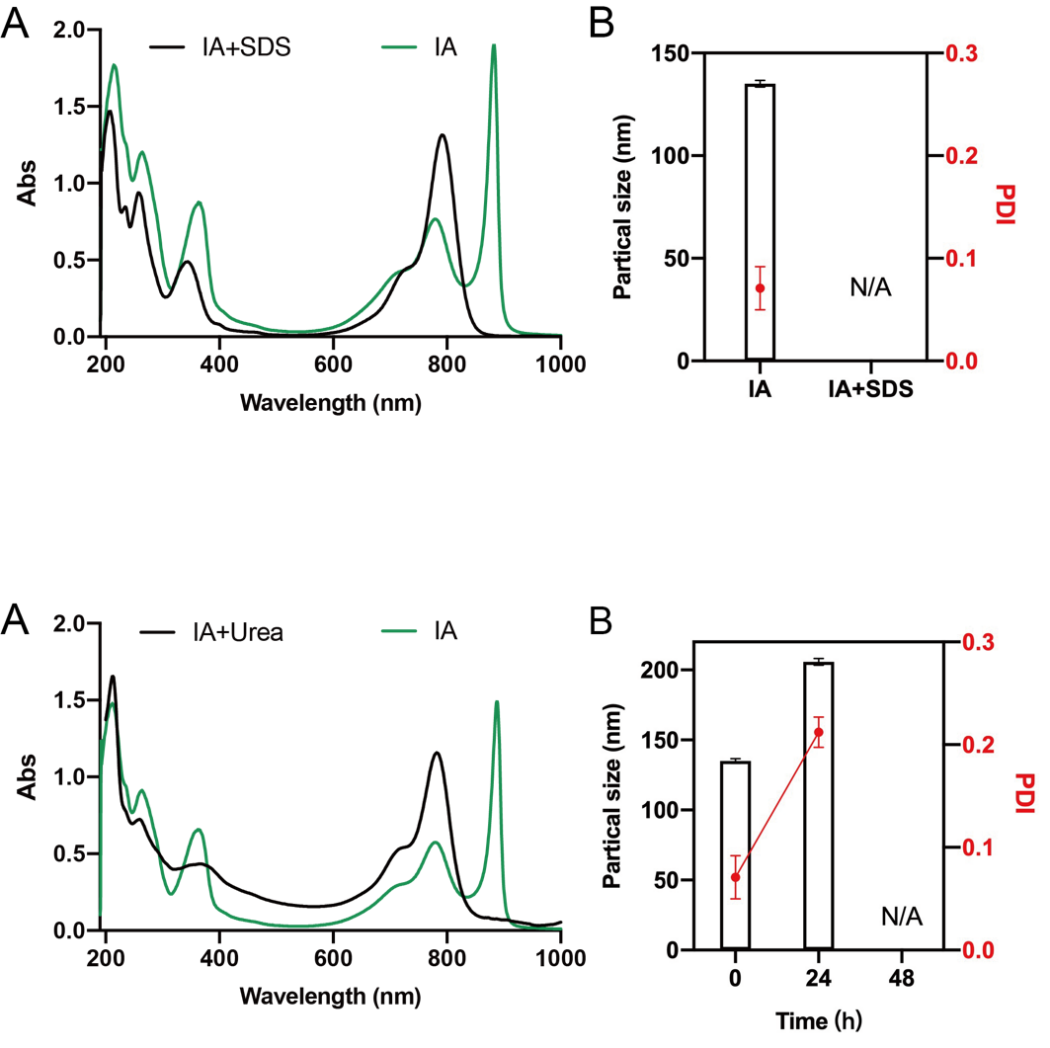


**Figure S4.** (A) UV-vis spectra of IA and IA+SDS. (B) The particle size and PDI changes of IA before and after SDS cultivation. All error bars indicate S.D. (n = 3).


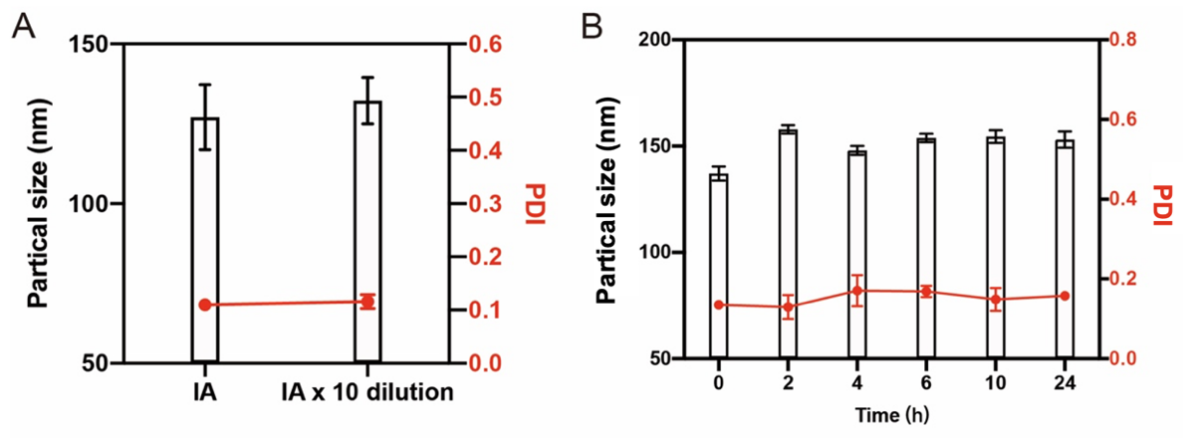


**Figure S5.** (A) The particle size of IA with 10 times dilution (25℃). (B) The particle size and PDI changes of IA incubated in 10% FBS within 24 h (37℃). All error bars indicate S.D. (n = 3).

**
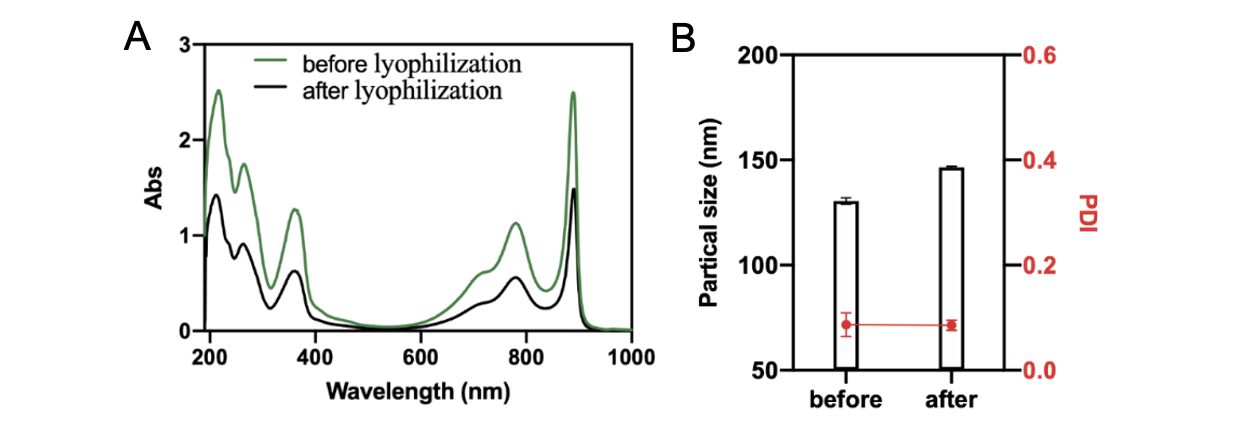
**

**Figure S6.** (A) UV-vis spectra of IA before and after lyophilization. (B) The particle size and PDI changes of IA before and after lyophilization. All error bars indicate S.D. (n = 3).


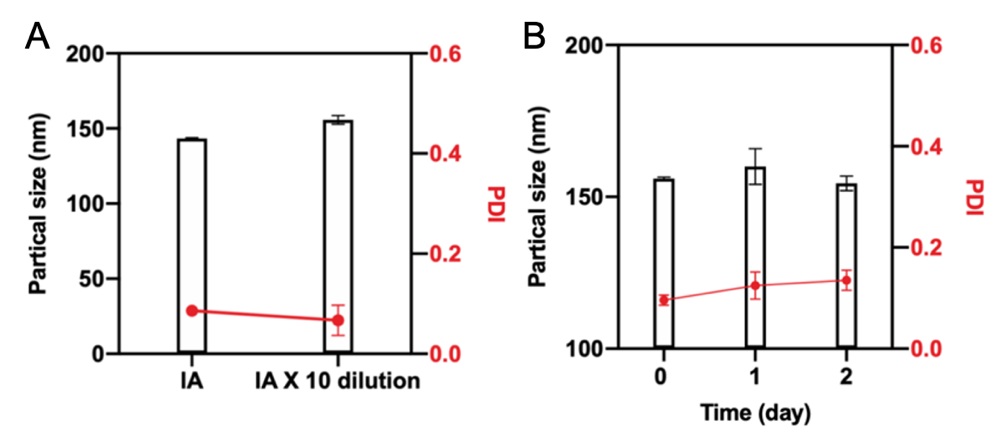


**Figure S7.** (A) The particle size and PDI of IA (after lyophilization) with 10 times dilution. (B) The particle size and PDI changes of IA solution (after lyophilization) within 2 days (25℃). All error bars indicate S.D. (n = 3).


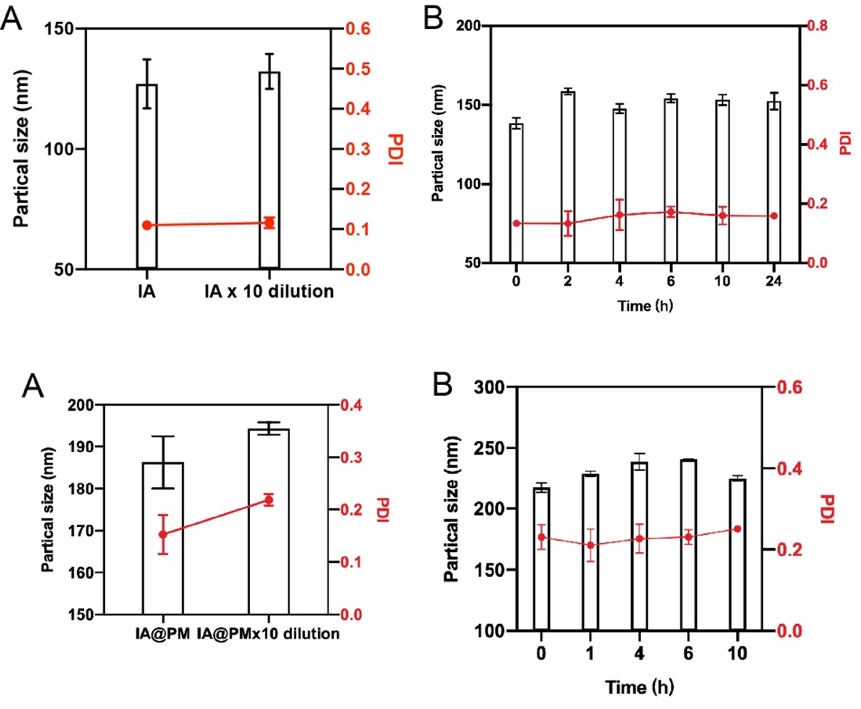


**Figure S8.** (A) The particle size and PDI changes of IA@PM with 10 times dilution (25℃). (B) The particle size and PDI changes of IA@PM incubated in 10% FBS within 10 h (37℃). All error bars indicate S.D. (n = 3).


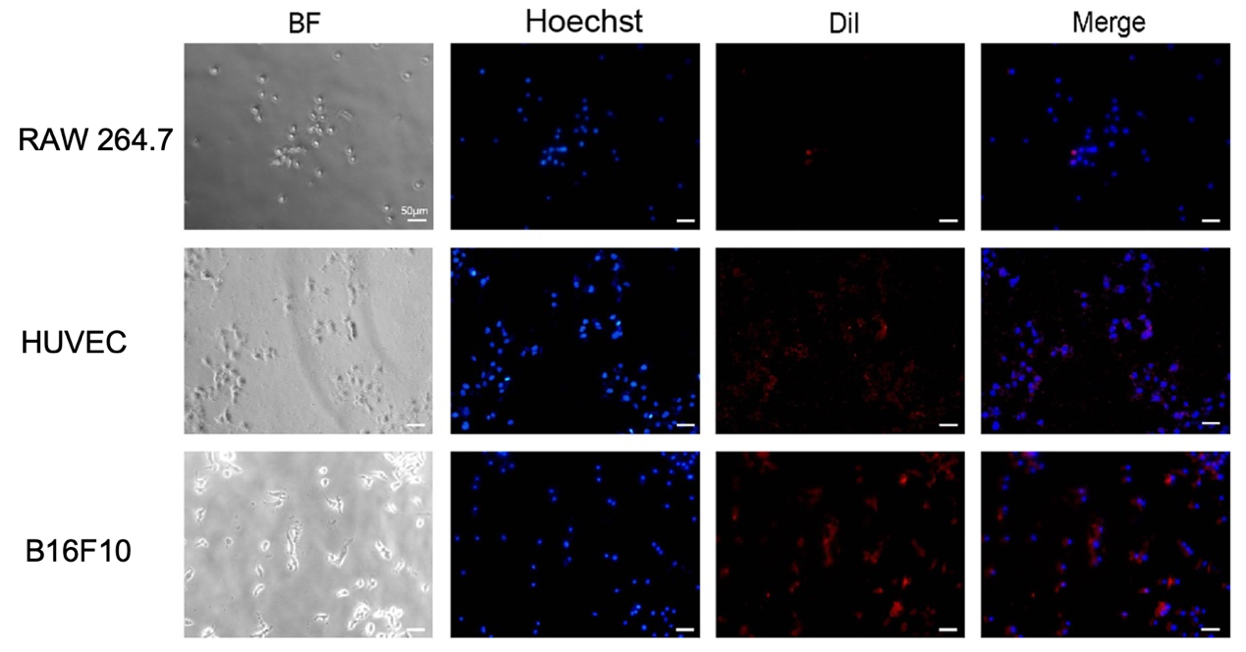


**Figure S9.** Converted microscope images of B16F10 cells, HUVEC and RAW 264.7 incubated with IA@PM. BF: bright field, red: DiI-labeled IA@PM, blue: Hoechst. Scale bar: 50 μm.


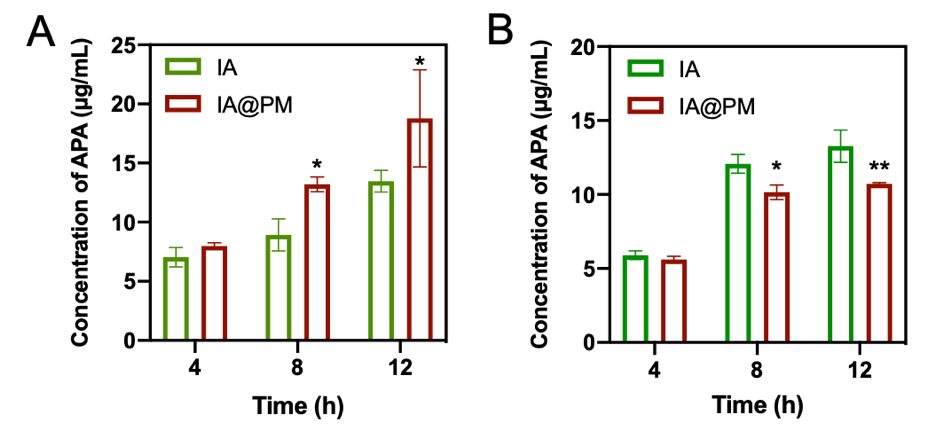


**Figure S10.** The concentration of APA in B16F10 cells (A) and RAW 264.7 (B) after 4 h, 8 h, and 12 h incubated with IA and IA@PM detected by HPLC, respectively. ^*^P <0.05, and ^**^P <0.01 *vs.* IA. All error bars indicate S.D. (n = 3).


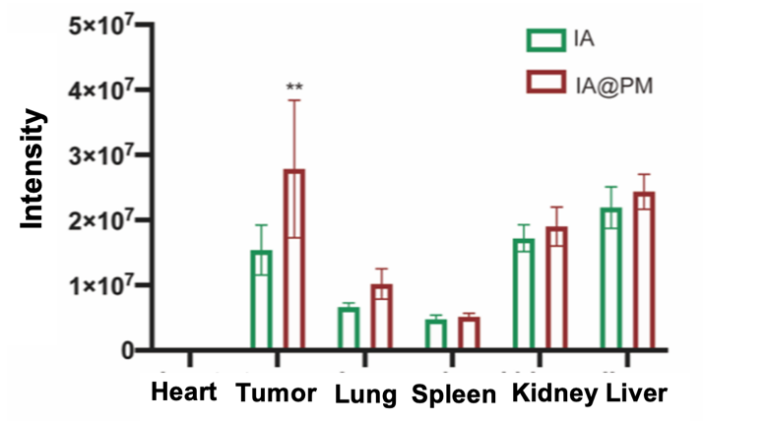


**Figure S11.** The average fluorescence intensity for the obtained tumors and organs administrated with IA and IA@PM. ^**^P <0.01 *vs.* IA. All error bars indicate S.D. (n = 3).


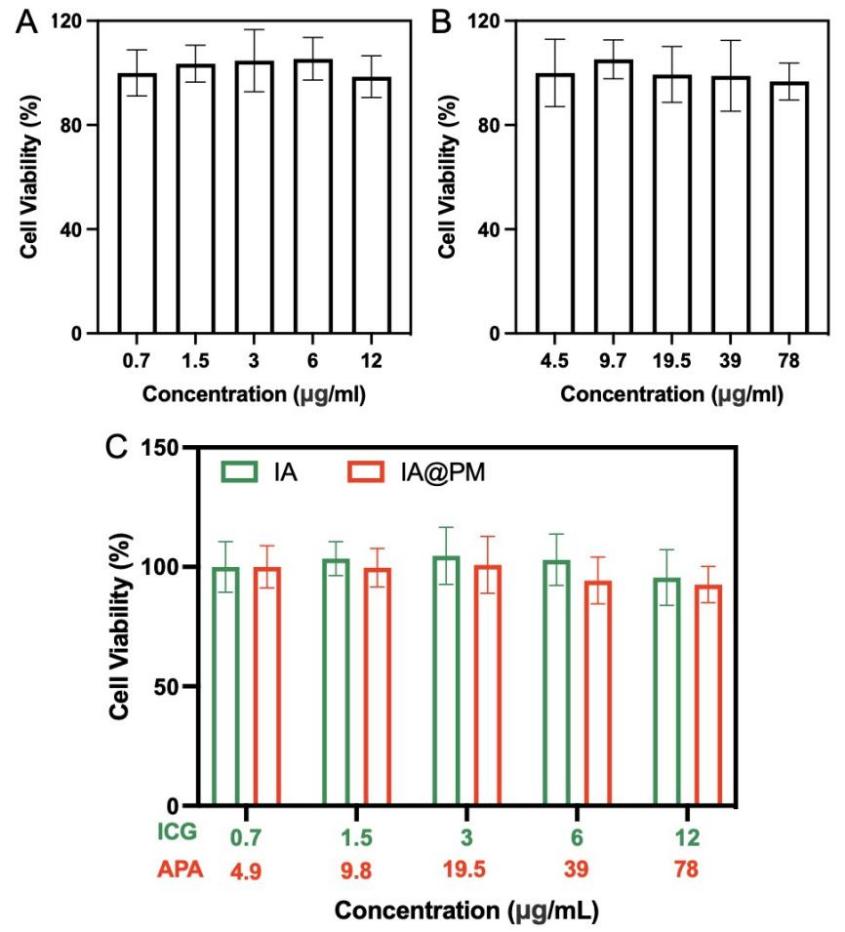


**Figure S12.** The cytotoxicity of ICG (A); APA (B); IA and IA@PM (C) against B16F10 cells. All error bars indicate S.D. (n = 6).


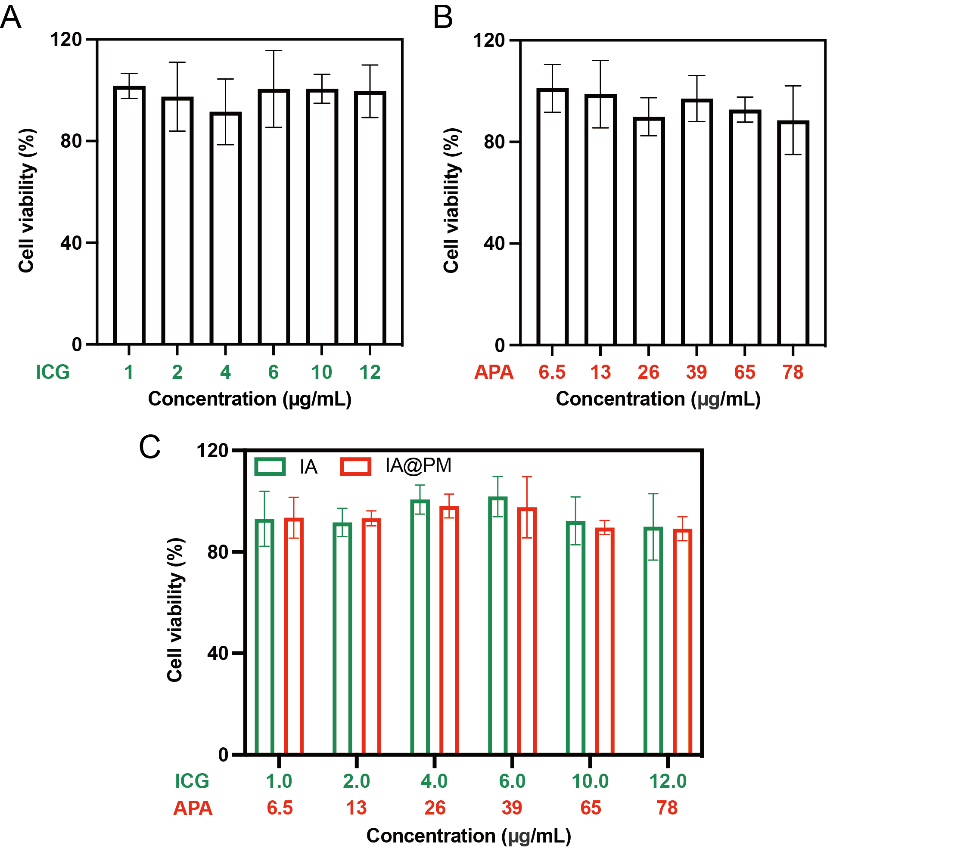


**Figure S13.** The cytotoxicity of ICG (A); APA (B); IA and IA@PM (C) against HUVEC. All error bars indicate S.D. (n = 6).


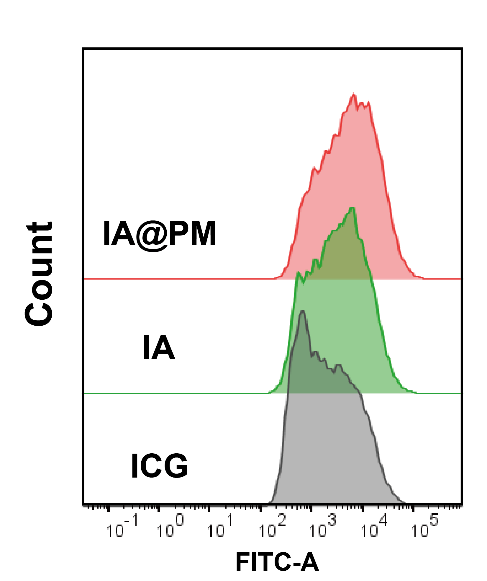


**Figure S14**. The flow cytometry image of ROS generation ability of ICG, IA, and IA@PM in B16F10 cells under 0.8 W cm^-2^ (2 min) laser after 12 h.


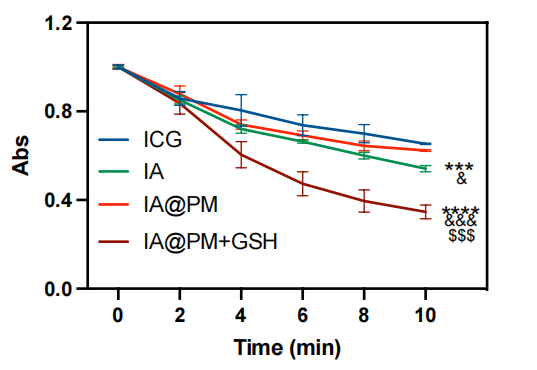


**Figure S15.** ROS production capacity of ICG, IA, IA@PM, and IA@PM+GSH under laser (808 nm, 0.8 W cm^-2^, 10 min). ^***^P <0.001, and ^****^P <0.0001 *vs.* ICG, ^&&^P <0.01, and ^&&&&^P <0.0001 *vs.* IA, ^$$$$^P <0.0001 *vs.* IA@PM. All error bars indicate S.D. (n = 3).


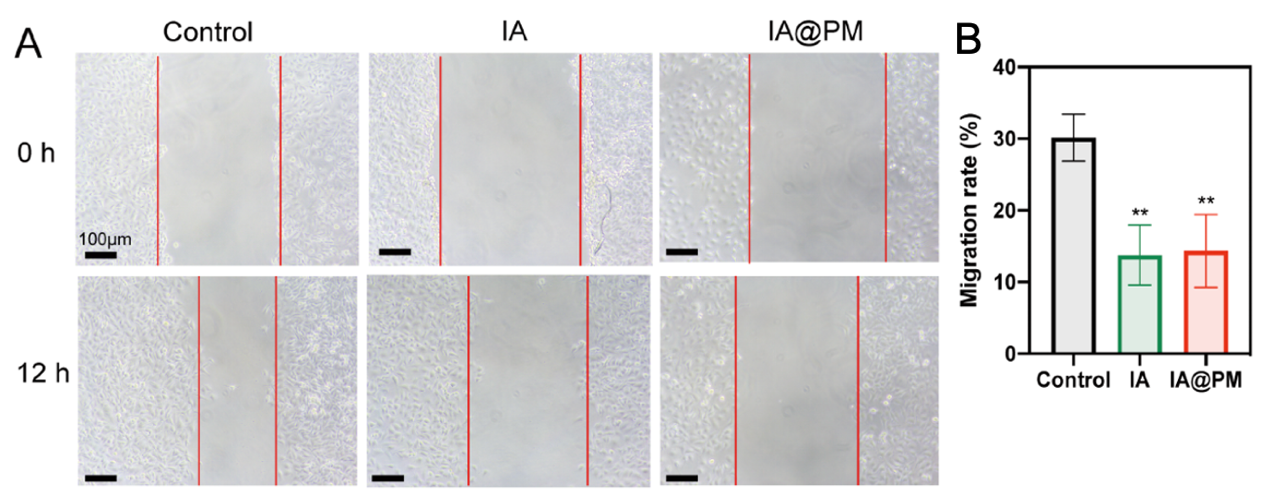


**Figure S16.** The cell migration images (A) and migration rates (B) of HUVEC incubated with IA and IA@PM at 0 h and 12 h (Scale bar: 100 μm). ^**^P <0.01 *vs.* Control. All error bars indicate S.D. (n = 3).


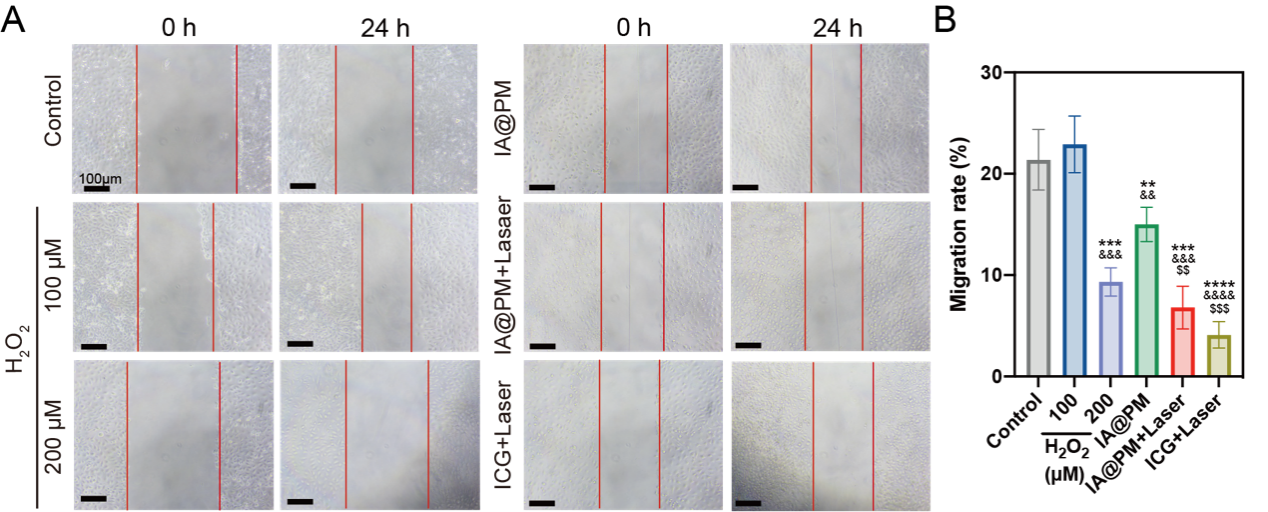


**Figure S17.** The cell migration images (A) and migration rates (B) of HUVEC incubated with H_2_O_2_ 100 μM, H_2_O_2_ 200 μM, IA@PM, IA@PM+Laser, and ICG+Laser at 0 h and 24 h (808 nm, 0.8 W cm^-2^, 5 min) (Scale bar: 100 μm). ^**^P <0.01, ^***^P <0.001, and ^****^P <0.0001 *vs.* Control; ^&&^P <0.01, ^&&&^P <0.001, and ^&&&&^P <0.0001 *vs.* H_2_O_2_ 100 μM; ^$$^P <0.01 and ^$$$^P <0.001 *vs.* IA@PM. All error bars indicate S.D. (n = 3).


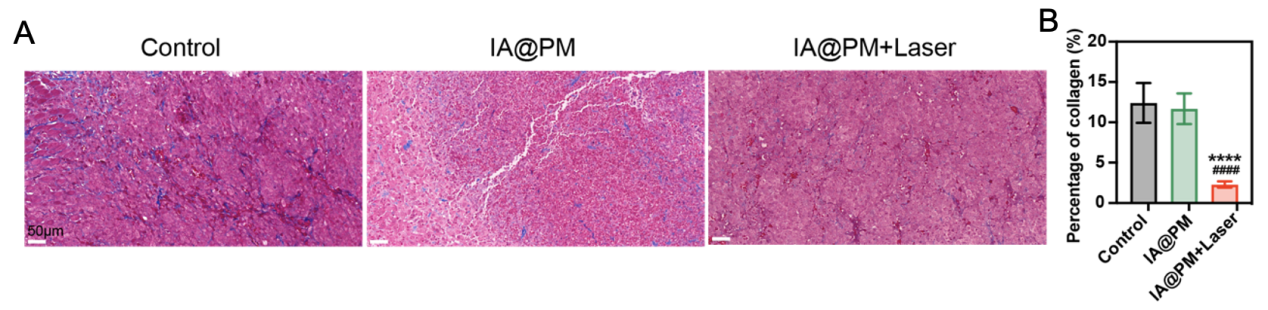


**Figure S18.** The images (A) and quantification statistics (B) of Masson staining (collagen) in tumor tissues from mice treated with saline, IA@PM, and IA@PM+Laser on day 4. Collagen was indicated in blue; cytoplasm was indicated in red (Scale bar: 50 μm). ^****^P <0.0001 *vs.* Control; ^####^P <0.0001 *vs.* IA@PM. All error bars indicate S.D. (n = 3).

**Figure S19.** The temperature of tumor after laser irradiation in ICG+Laser, IA+Laser, and IA@PM+Laser groups (808 nm, 0.6 W cm^-2^, 5 min).


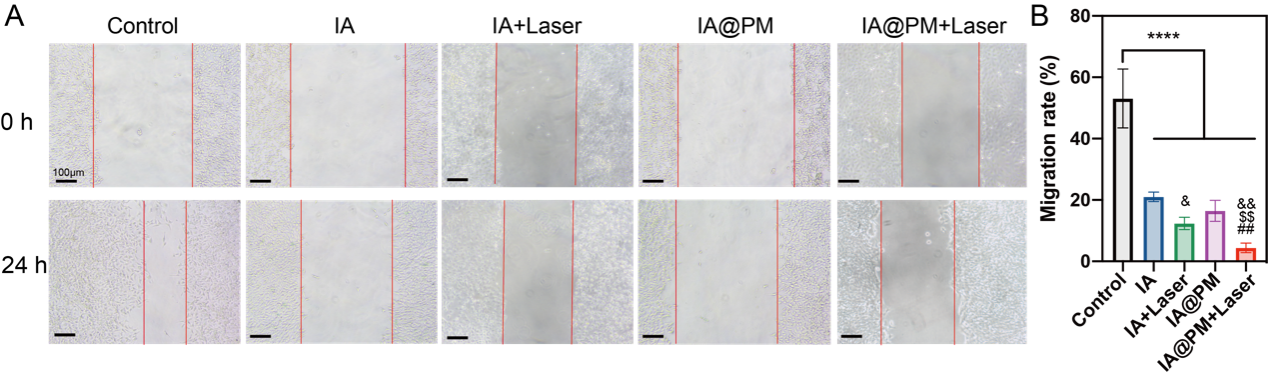


**Figure S20.** The cell migration images (A) and migration rates (B) of B16F10 cells treated with IA, IA+Laser, IA@PM, and IA@PM+Laser at 0 h and 24 h (808 nm, 0.8 W cm^-2^, 5 min) (Scale bar: 100 μm). ^****^P <0.0001 *vs.* Control; ^&^P <0.05 and ^&&^P <0.01 *vs.* IA; ^$$^P <0.01 *vs.* IA+Laser; ^##^P <0.01 *vs.* IA@PM. All error bars indicate S.D. (n = 3).


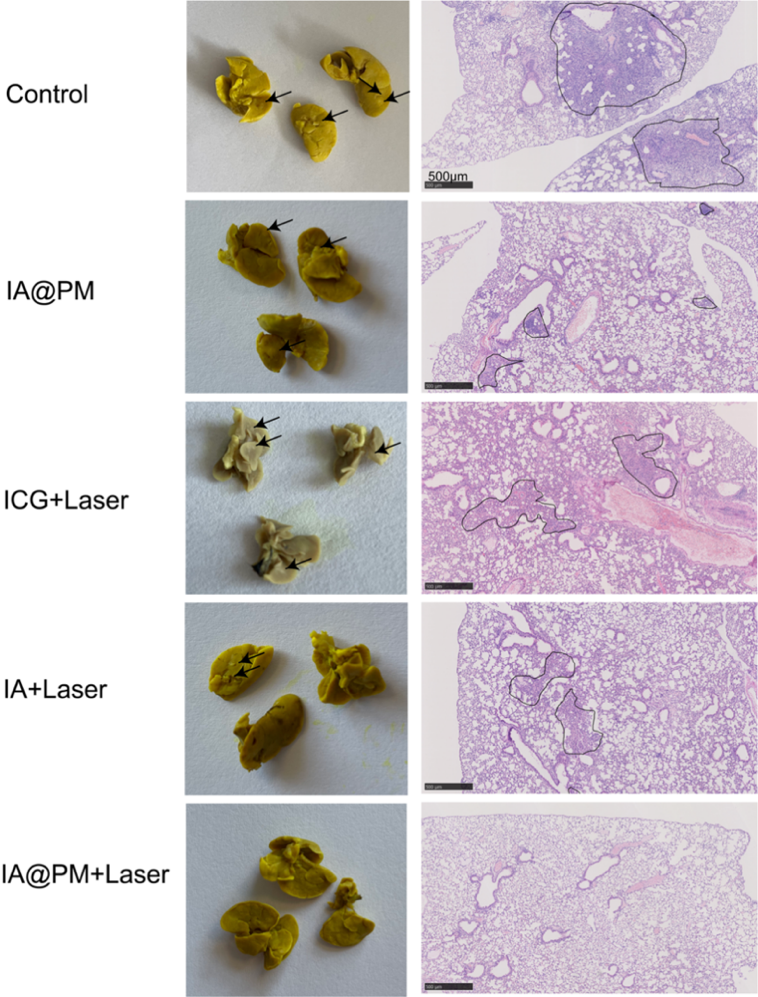


**Figure S21.** Bouin’s staining buffer-treated lungs with metastasis nodules from subcutaneous melanoma model and H&E staining images shown the metastasis region in lungs. Black arrows and circles indicate tumor nodules. Scale bar: 500 μm.


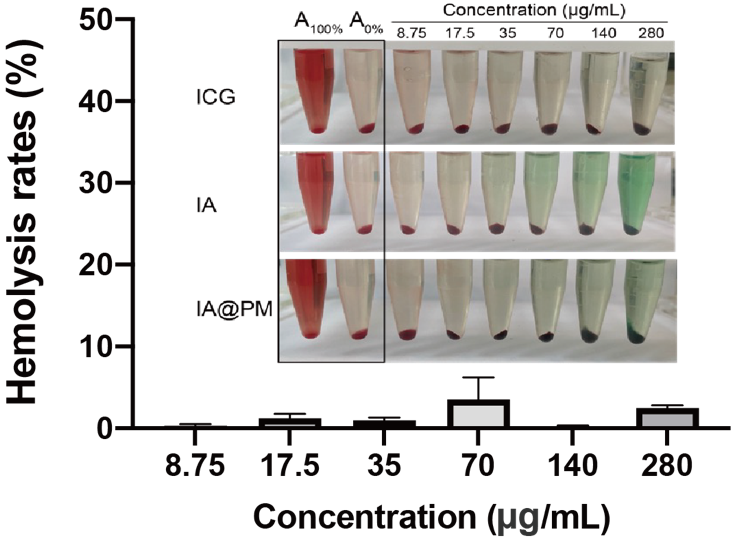


**Figure S22.** The hemolysis rates of IA@PM. All error bars indicate S.D. (n = 3).


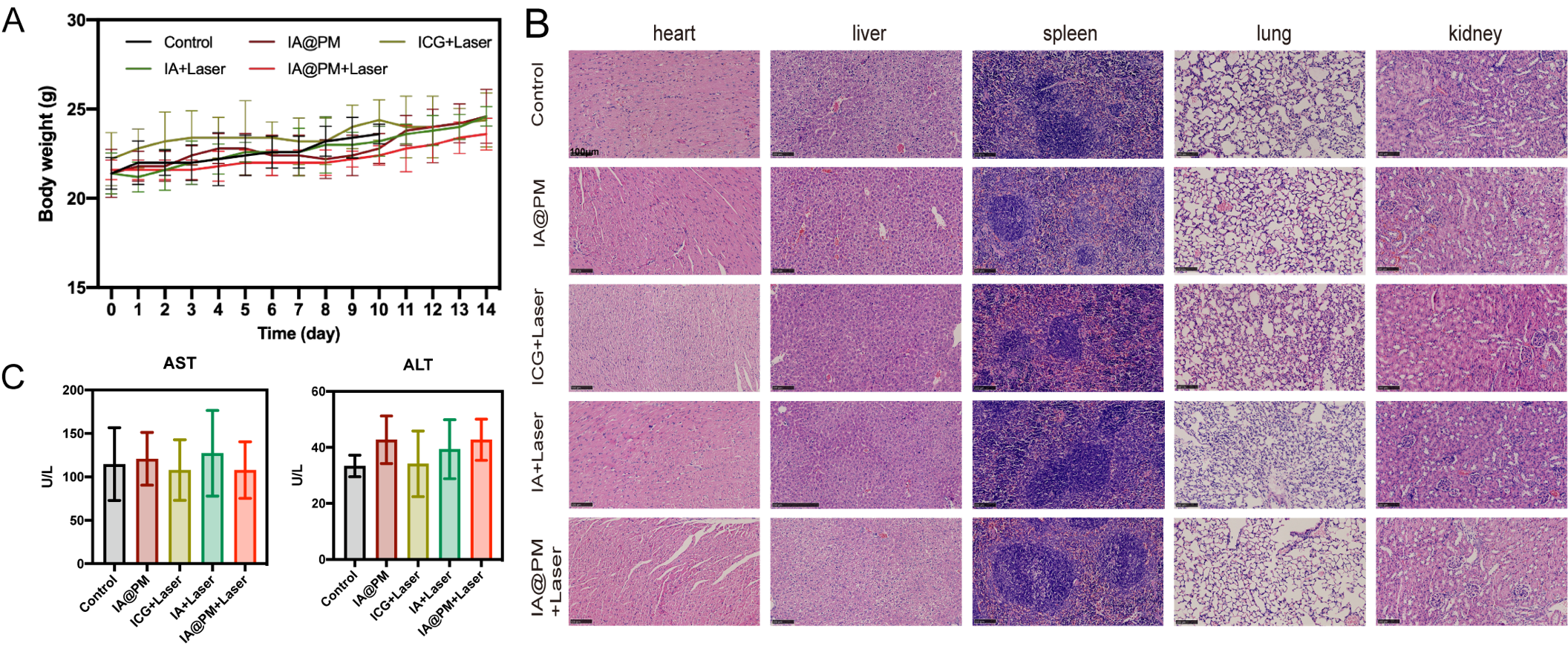


**Figure S23.** The body weight changes of B16F10 tumor-bearing mice with different treatments. All error bars indicate S.D. (n = 5).


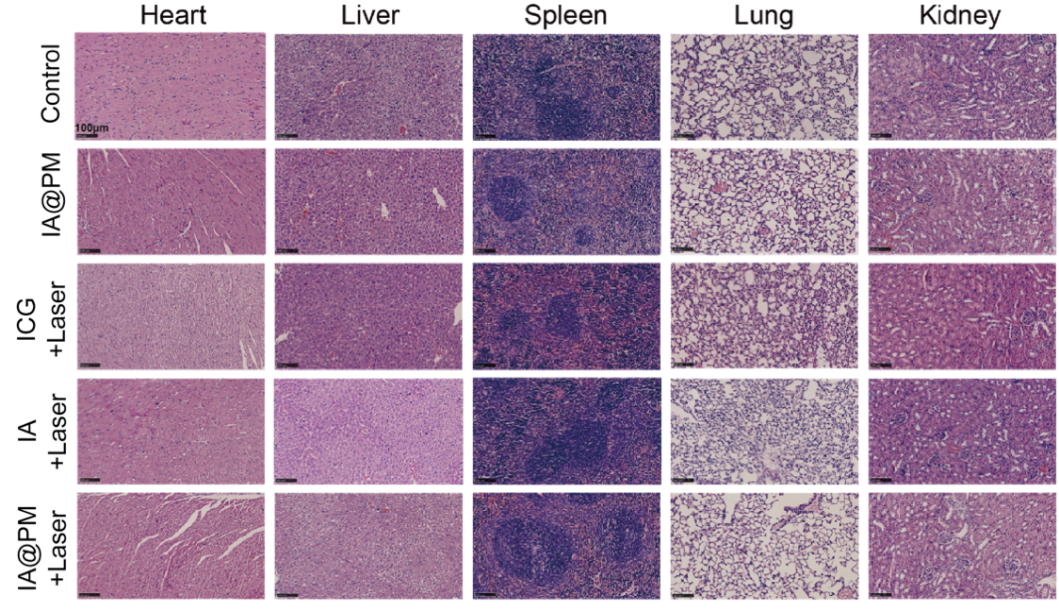


**Figure** **S24.** The H&E staining images of heart, liver, spleen, lung, and kidney. Scale bar: 100 μm.


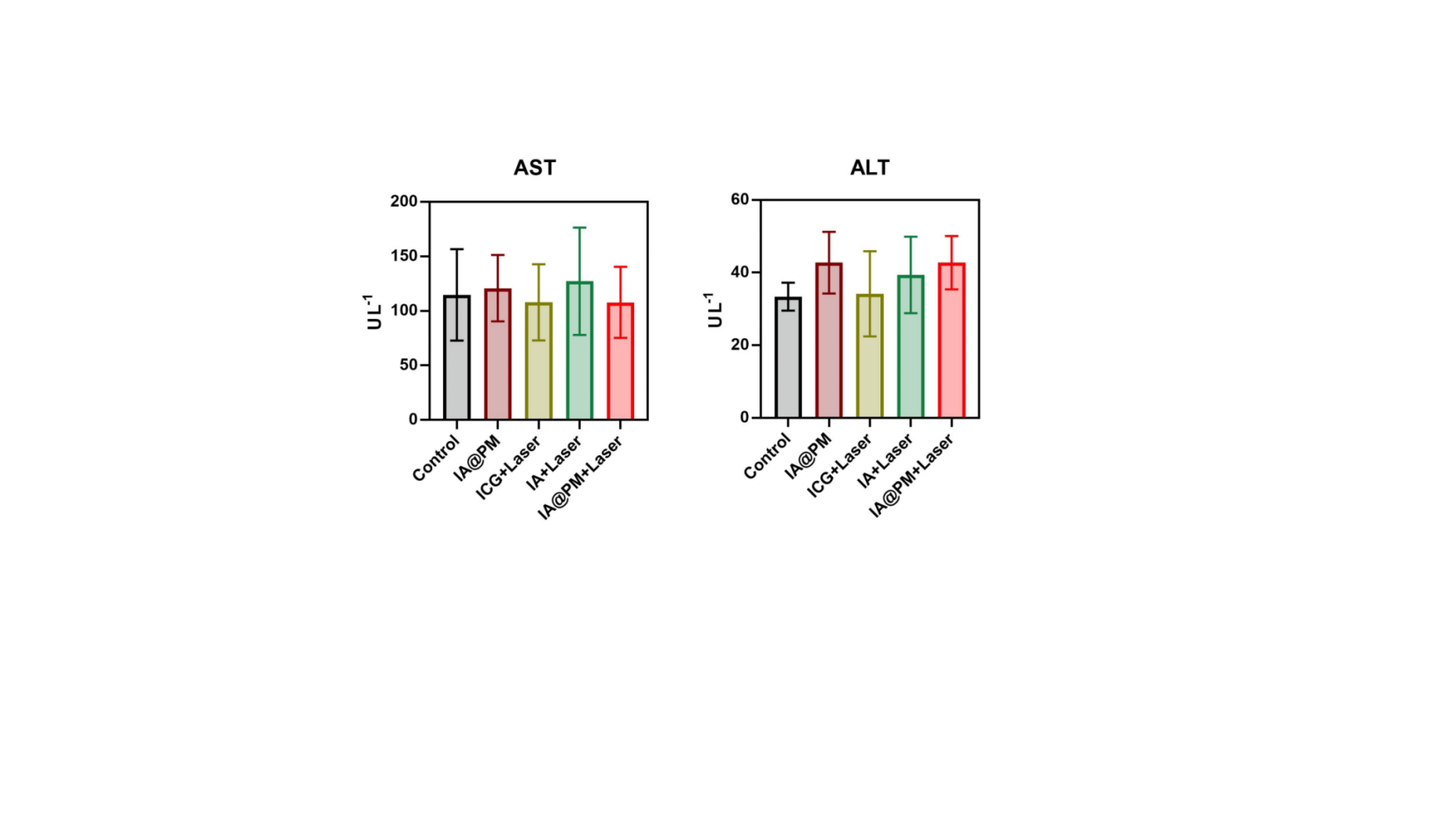


**Figure S25.** Blood biochemistry analysis (ALT and AST) of the tumor-bearing mice treated with different formulations. All error bars indicate S.D. (n = 5).

**Table S1.** Particle size, PDI, and zeta potential of IA and DL of ICG and APA from different batches (n = 3).

| Batch | Particle size (nm) | PDI | Zeta potential (mV) | DL (%) | |
| --- | --- | --- | --- | --- | --- |
|  |  |  |  | ICG | APA |
| 1# | 126.00±0.91 | 0.053±0.011 | -29.63±0.46 | 14.63±0.51 | 85.37±0.51 |
| 2# | 129.89±1.74 | 0.048±0.013 | -34.54±1.75 | 12.05±0.42 | 87.95±0.42 |
| 3# | 135.32±2.18 | 0.024±0.023 | -30.03±0.76 | 13.07±0.77 | 86.93±0.77 |

**Table S2.** Characteristic FTIR peak of ICG, APA, IA, and APA+ICG. (N.A. means not applicable.)

| Wavenumber range | ICG | APA | IA | APA+ICG |
| --- | --- | --- | --- | --- |
| >2000 cm^-1^ | 3339.4 cm^-1^ | N.A. | 3351.2 cm^-1^ | N.A. |
| <2000 cm^-1^ | N.A. | 1658.1-1638.6 cm^-1^ | 1589.7-1574.3 cm^-1^ | N.A. |
|  | 1509.2 cm^-1^ | N.A. | 1514.1 cm^-1^ | 1508.7 cm^-1^ |

**Table S3.** The significant differences of tumor volume after treated with different administration on 10^th^ day (n = 5). (N.A. means not applicable, and N.S. means no significant.)

|  | IA@PM | ICG+Laser | IA+Laser | IA@PM+Laser |
| --- | --- | --- | --- | --- |
| Control | P<0.0001 | P<0.0001 | P<0.0001 | P<0.0001 |
| IA@PM | N.A. | N.S. | P<0.05 | P<0.001 |
| ICG+Laser | N.A. | N.A. | N.S. | P<0.01 |
| IA+Laser | N.A. | N.A. | N.A. | P<0.01 |
